# Safety Evaluation of α-Lipoic Acid Supplementation: A Systematic Review and Meta-Analysis of Randomized Placebo-Controlled Clinical Studies

**DOI:** 10.3390/antiox9101011

**Published:** 2020-10-19

**Authors:** Federica Fogacci, Manfredi Rizzo, Christoffer Krogager, Cormac Kennedy, Coralie M.G. Georges, Tamara Knežević, Evangelos Liberopoulos, Alexandre Vallée, Pablo Pérez-Martínez, Eliane F.E. Wenstedt, Agnė Šatrauskienė, Michal Vrablík, Arrigo F.G. Cicero

**Affiliations:** 1 Hypertension and Cardiovascular Risk Factors Research Group, Medical and Surgical Sciences Department, Sant’Orsola-Malpighi University Hospital, 40138 Bologna, Italy; federica.fogacci@studio.unibo.it; 2 Department of Health Promotion Sciences Maternal and Infantile Care, Internal Medicine and Medical Specialities (PROMISE), School of Medicine, University of Palermo, 90127 Palermo, Italy; manfredi.rizzo@unipa.it; 3 Department of Endocrinology, Aarhus University Hospital, DK-8200 Aarhus N, Denmark; chrkroga@rm.dk; 4 Department of Pharmacology and Therapeutics, Trinity College Dublin and St James Hospital, Dublin 8, Ireland; kennec30@tcd.ie; 5 Department of Cardiology, Cliniques Universitaires Saint-Luc, Université Catholique de Louvain, 1200 Brussels, Belgium; coralie.georges@uclouvain.be; 6 Department of Nephrology, Hypertension, Dialysis and Transplantation, University Hospital Centre Zagreb, 10 000 Zagreb, Croatia; tknezev2@kbc-zagreb.hr; 7 Faculty of Medicine, School of Health Sciences, University of Ioannina, 451 10 Ioannina, Greece; elibero@uoi.gr; 8 Diagnosis and Therapeutic Center, Hôtel-Dieu Hospital, Paris-Descartes University, 75004 Paris, France; alexandre.g.vallee@gmail.com; 9 CIBER Fisiopatología de la Obesidad y Nutrición (CIBEROBN), Instituto de Salud Carlos III (ISCIII), 28007 Madrid, Spain; pabloperez@uco.es; 10 Lipids and Atherosclerosis Unit, Department of Internal Medicine, Reina Sofia University Hospital, 14004 Cordoba, Spain; 11 Maimonides Biomedical Research Institute of Cordoba (IMIBIC), 14004 Cordoba, Spain; 12 Department of Medicine (Medicine, Dermatology and Otorhinolaryngology), University of Cordoba, 14004 Cordoba, Spain; 13 Amsterdam UMC—University of Amsterdam, 1100 DD Amsterdam, The Netherlands; elianewenstedt@live.nl; 14 Faculty of Medicine, Vilnius University, LT-03101 Vilnius, Lithuania; agne.satrauskiene@santa.it; 15 Vilnius University Hospital Santariškių Klinikos, LT-08661 Vilnius, Lithuania; 16 Third Department of Internal Medicine, First Medical Faculty, Charles University, 128 08 Prague 2, Czech Republic; vrablikm@seznam.cz

**Keywords:** α-lipoic acid, thioctic acid, dietary supplement, safety, meta-analysis

## Abstract

Alpha-lipoic acid (ALA) is a natural short-chain fatty acid that has attracted great attention in recent years as an antioxidant molecule. However, some concerns have been recently raised regarding its safety profile. To address the issue, we aimed to assess ALA safety profile through a systematic review of the literature and a meta-analysis of the available randomized placebo-controlled clinical studies. The literature search included EMBASE, PubMed Medline, SCOPUS, Google Scholar, and ISI Web of Science by Clarivate databases up to 15th August 2020. Data were pooled from 71 clinical studies, comprising 155 treatment arms, which included 4749 subjects with 2558 subjects treated with ALA and 2294 assigned to placebo. A meta-analysis of extracted data suggested that supplementation with ALA was not associated with an increased risk of any treatment-emergent adverse event (all *p* > 0.05). ALA supplementation was safe, even in subsets of studies categorized according to smoking habit, cardiovascular disease, presence of diabetes, pregnancy status, neurological disorders, rheumatic affections, severe renal impairment, and status of children/adolescents at baseline.

## 1. Introduction

Alpha-lipoic acid (1, 2-dithiolane-3-pentanoic acid; ALA) or thioctic acid is a natural short-chain fatty acid that has attracted great attention in recent years as an antioxidant molecule, being largely used worldwide as a dietary supplement [1].

Previous investigations revealed that ALA can affect central and peripheral modulation of 5′-adenosine-monophosphate-activated protein kinase. Furthermore, it activates peroxisome proliferator-activated receptor (PPAR) alpha and gamma (PPAR-γ), modulates PPAR-regulated genes and upregulates the expression of PPAR-γ messenger ribonucleic acid (mRNA) and other proteins in the cardiac tissue and aorta smooth muscle [2,3]. Hence, ALA antioxidant activity is potentially able to promote weight loss and blood pressure control and ameliorate atherogenic dyslipidemia and insulin resistance [3]. For example, in obese patients with non-alcoholic fatty liver disease (NAFLD), ALA supplementation was shown to reduce adipokine concentrations and improve liver steatosis grade [4,5]. However, some concerns have been recently raised regarding ALA safety profile, after some reports suggesting a direct causal link between its use and insulin autoimmune syndrome (IAS, also known as Hirata’s disease) due to its sulfhydryl group [6]. Indeed, in about 50% of cases, IAS development is associated with drugs or dietary supplement containing a sulphur or sulfhydryl group. These cases are closely related to certain specific antigens of the major histocompatibility complex (MHC), which are more common in populations where IAS incidence is higher [7]. It is hypothesised that ALA might cause the development of antibodies to insulin and lead to a hypoglycaemic syndrome in predisposed subjects, even though evidence are inconclusive [8].

In a recent study that performed a preliminary analysis of spontaneous reports of suspected adverse reactions (ARs), ALA-containing natural products have also been associated with skin and gastrointestinal disorders, such as urticaria and abdominal pain [9].

To address safety issues related to ALA supplementation, we aimed to perform a systematic review of the literature and a meta-analysis of the available randomized placebo-controlled clinical trials.

## 2. Materials and Methods

The study was designed according to guidelines of the 2009 preferred reporting items for systematic reviews and meta-analysis (PRISMA) statement [10], and was registered in the PROSPERO database (Registration number CRD42020159028).

Due to the study design, neither Institutional Review Board (IRB) approval, nor patient informed consent were required. PRISMA Checklist was reported in supplementary file A.

### 2.1. Search Strategy

EMBASE, PubMed Medline, SCOPUS, Google Scholar and ISI Web of Science by Clarivate databases were searched, with no language restriction, using the following search terms: (“Alpha-lipoic acid” OR “Alpha lipoic acid” OR “α-lipoic acid” OR “α lipoic acid” OR “ALA” OR “A-LA” OR “Lipoic acid” OR “Thioctic acid” OR “Tioctic acid” OR “Thioctacid”) AND (“Clinical trial” OR “Clinical study”). The wild-card term “*” was used to increase the sensitivity of the search strategy, which was limited to studies in humans. The reference list of identified papers was manually checked for additional relevant articles. Additional searches included references of review articles on that issue, and abstracts from selected congresses on the subject of the meta-analysis. Literature was searched from inception to 15th August 2020.

All paper abstracts were firstly screened by two independent reviewers (F.F. and M.R.) to remove ineligible articles. The remaining articles were obtained in full-text and assessed again by the same two researchers who evaluated each article independently and carried out data extraction and quality assessment. Disagreements were resolved by discussion with a third party (A.F.G.C.).

### 2.2. Study Selection Criteria

Original studies were included if they met the following criteria: (i) being a clinical trial with either parallel or cross-over design, (ii) having an appropriate controlled design for ALA supplementation, (iii) blinding participants to intervention, (iv) testing the safety of ALA, (v) reporting treatment-emergent adverse events.

Exclusion criteria were: (i) lack of randomisation for treatment allocation, (ii) lack of a control group receiving placebo (iii) lack of sufficient information about the prevalence and nature of the adverse events. Studies were also excluded if they contained overlapping subjects with other studies.

### 2.3. Data Extraction

Data abstracted from eligible studies were: (i) first author’s name; (ii) year of publication; (iii) study location; (iv) study design; (v) follow-up; (vi) main inclusion criteria and underlying disease; (vii) study groups; (viii) number of participants in the active and control group; (ix) age and sex of study participants; (x) treatment-emergent adverse events occurred during the trials. Missing or unpublished data were sought by trying to contact authors via e-mail and repeated messages were sent in case of no response. Extracted data were reviewed by the principal investigator before the final analysis, and doubts were resolved by mutual agreement among the authors.

### 2.4. Quality Assessment

A systematic assessment of risk of bias in the included studies was performed using the Cochrane criteria [11]. The following items were used: adequacy of sequence generation, allocation concealment, blinding addressing of dropouts (incomplete outcome data), selective outcome reporting, and other probable sources of bias [12]. Overall evidence was qualified using the Grading of Recommendations, Assessment, Development, and Evaluations (GRADE) system [13]. Risk-of-bias assessment was performed independently by two reviewers; disagreements were resolved by a consensus-based discussion.

### 2.5. Data Synthesis

Meta-analysis was conducted using Comprehensive Meta-Analysis (CMA) V3 software (Biostat, NJ) [14].

Outcomes were treatment-emergent adverse events (AEs) occurring during the trials. In particular, data extracted from the studies included hypoglycaemic episodes, gastrointestinal AEs (e.g., heartburn, gastric complaints, nausea, gastrointestinal complications, duodenitis, and abdominal bloating), neurological AEs (e.g., headache, foggy thinking, drowsiness, leg weakness, legs periodic numbness and tingling, tingling in toe and fingers and intermittent bilateral toe numbness), psychiatric disorders (e.g., bipolar disorders, irritability, poor sleeping), musculoskeletal AEs (e.g., neck pain, lower back pain, and spasms), skin AEs (e.g., skin rash, disseminated maculopapular rash, itching sensation and urticaria), infections (e.g., laryngitis, pneumonia and yeast infections), cardiovascular (CV) system AEs (e.g., increase in arterial blood pressure, palpitations, myocardial infarction, heart rate and rhythm disorders, and heart valve disorders), hospitalisation and death.

The analysis was performed by excluding studies with zero events in both arms. If one or more outcomes could not be extracted from a study, the study was removed only from the analysis involving those outcomes. To avoid a double-counting problem, in trials comparing multiple treatment arms versus a single control group, the number of subjects in the control group was divided by the required comparisons [15].

To reduce the risk of bias due to effect dilution, the meta-analysis was performed considering per-protocol (PP) population.

Studies’ findings were combined using a fixed-effect model since the low level of inter-study heterogeneity, which was quantitatively assessed using the Higgins index (I^2^) [16]. Effect sizes were expressed as odds ratio (OR) and 95% confidence interval (95% CI) [17]. Finally, sensitivity analysis was conducted to account for the risk of bias. A leave-one-out method was used (i.e., one study was removed at a time and the analysis was repeated) [18].

Two-sided *p*-values < 0.05 were considered as statistically significant for all tests.

### 2.6. Additional Analysis

Subgroup analyses were carried out by presence of smoking habit, pregnancy, CV disease, diabetes, rheumatic disorders, neurological disorders, severe renal impairment, and status of children/adolescent at baseline.

### 2.7. Publication Biases

Potential publication biases were explored using visual inspection of Begg’s funnel plot asymmetry, Begg’s rank correlation test, and Egger’s weighted regression test [19]. Two-sided *p*-values < 0.05 were considered statistically significant for the tests.

## 3. Results

### 3.1. Flow and Characteristics of the Included Studies

**Figure 1:**
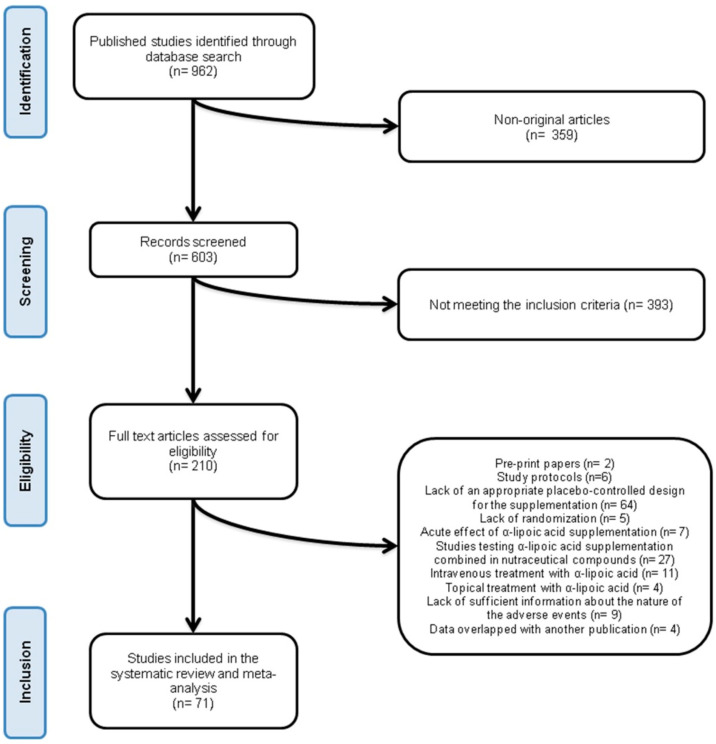
Flow chart of the number of studies identified and included in the systematic review.

After database searches performed strictly according to inclusion and exclusion criteria, 962 published articles were identified, and their abstracts reviewed. Of these, 359 did not report original data. Furthermore, 393 articles were excluded because they did not meet the inclusion criteria. Thus, 210 articles were carefully assessed and reviewed. Additional 139 papers were excluded due to being pre-print papers (*n* = 2), study protocols (*n* = 6), reporting data from studies lacking of an appropriate placebo-controlled design for the supplementation (*n* = 64), lacking of randomisation (*n* = 5), testing the acute effect of ALA supplementation (*n* = 7), testing ALA supplementation combined in nutraceutical compounds (*n* = 27), testing intravenous treatment with ALA (*n* = 11), testing topical treatment with ALA (*n* = 4), lacking sufficient information about the nature of the adverse events (*n* = 9), or reporting data overlapped with other publications (*n* = 4) (Supplementary file B). Finally, 71 studies were eligible and included in the systematic review [20,21,22,23,24,25,26,27,28,29,30,31,32,33,34,35,36,37,38,39,40,41,42,43,44,45,46,47,48,49,50,51,52,53,54,55,56,57,58,59,60,61,62,63,64,65,66,67,68,69,70,71,72,73,74,75,76,77,78,79,80,81,82,83,84,85,86,87,88,89,90]. The study selection process is shown in Figure 1.

Data were pooled from 71 randomized placebo-controlled clinical studies, comprising 155 treatment arms (82 active arms and 73 control arms). The studies included 4749 subjects, with 2558 receiving treatment with ALA and 2294 subjects assigned to placebo. For reasons independent of the tested supplementation (i.e., withdrawal of informed consent and personal problems), 510 subjects prematurely terminated the trials in which they were enrolled. Then, the meta-analysis was performed considering the other subjects (i.e., PP population).

Eligible studies were published between 1982 and 2020 and were conducted in different locations across all continents. Follow-up periods ranged between 8 days and 4 years and several ALA regimens were tested. Selected clinical trials were designed with cross-over or parallel-group and enrolled pregnant women with gestational diabetes, children and/or adolescent, overall healthy subjects or subjects with minor or major underlying diseases (e.g., diabetes, CVD, rheumatic affections, neurological disorders, severe renal impairment).

Included clinical studies were fully or partially carried out independently and funded by the National Institutes of Health (*n* = 7), Health Ministries (*n* = 2), University Institutes (*n* = 42), Research Hospitals (*n* = 2), Private Research Institutes (*n* = 2), Scientific Societies (*n* = 3), Private Foundations (*n* = 8), or were financially supported by industries (*n* = 7).

The main characteristics of the evaluated studies are summarized in Table 1.

### 3.2. Risk of Bias Assessment

Almost all of the included studies were characterized by sufficient information regarding sequence generation, allocation concealment, personal and outcome assessments, incomplete outcome data, and selective outcome reporting. Details of the quality of bias assessment are reported in Table 2.

The quality of evidence for each outcome across all the studies was considered high in accordance with the GRADE approach.

### 3.3. Primary Outcomes

#### 3.3.1. Hypoglycaemic Episodes

Symptoms defined as ‘similar to hypoglycaemic episodes’ were reported only by Jacob et al. and were exclusively experienced by subjects randomized to placebo. Authors did not report if an attempt for treatment rechallenging was made during the trial [44].

#### 3.3.2. Gastrointestinal AEs

**Figure 2:**
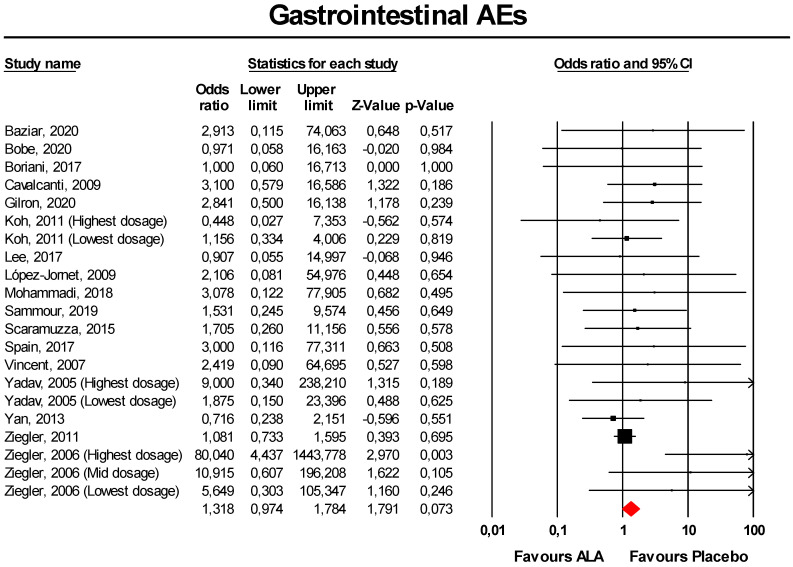
Forest plot for the risk of gastrointestinal adverse events (AEs) following alpha-lipoic acid (ALA) supplementation *versus* placebo.

Meta-analysis of extracted data suggested that supplementation with ALA was not associated with an increased risk of gastrointestinal AEs (OR = 1.32, 95% CI 0.97 to 1.78; *p* = 0.073; I^2^ = 0%) (Figure 2). The finding was robust in the leave-one-out sensitivity analysis (Figure S1).

Visually, the funnel plot of standard error by log OR was slightly asymmetric (Figure S2). This asymmetry was imputed to eight potentially missing studies on the left-side of the plot, which reduced the estimated effect size to 1.12 (95% CI 0.84 to 1.49). Egger’s linear regression and Begg’s rank correlation confirmed the presence of publication bias for the analysis (*p* < 0.05).

#### 3.3.3. Neurological AEs

**Figure 3:**
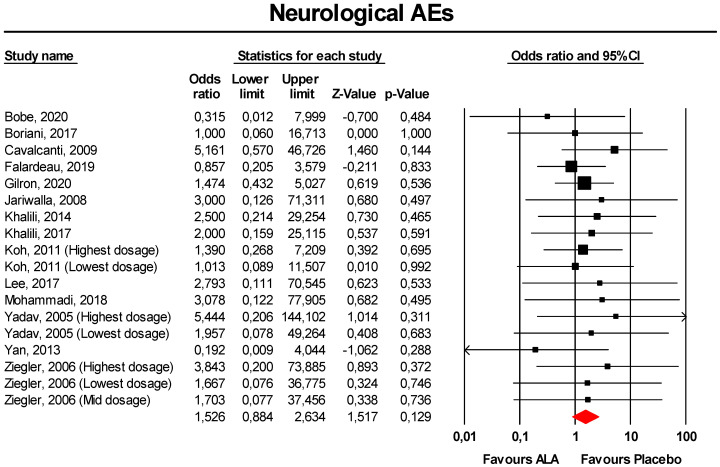
Forest plot for the risk of neurological AEs following ALA supplementation *versus* placebo.

Meta-analysis of extracted data suggested that supplementation with ALA was not associated with an increased risk of neurological AEs (OR = 1.53, 95% CI 0.88 to 2.63; *p* = 0.129; I^2^ = 0%) (Figure 3). The finding was robust in the leave-one-out sensitivity analysis (Figure S3).

Visually, the funnel plot of standard error by log OR was slightly asymmetric (Figure S4). This asymmetry was imputed to 4 potentially missing studies on the left-side of the plot, which reduced the estimated effect size to 1.26 (95% CI 0.76 to 2.10). However, neither Egger’s linear regression nor Begg’s rank correlation confirmed the presence of publication bias for the analysis (*p* > 0.05 for both tests).

#### 3.3.4. Psychiatric Disorders

**Figure 4:**
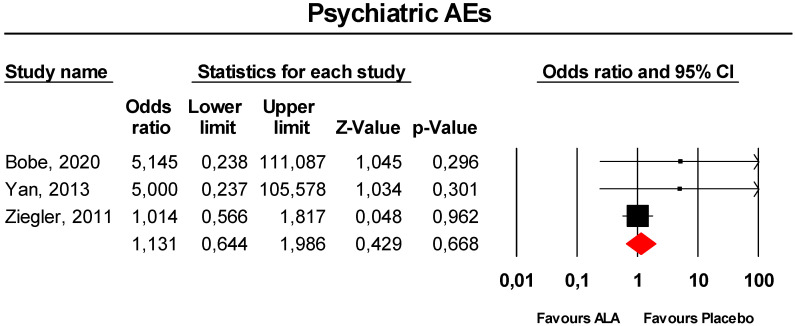
Forest plot for the risk of psychiatric AEs following ALA supplementation *versus* placebo.

Meta-analysis of extracted data suggested that supplementation with ALA was not associated with an increased risk of psychiatric disorders (OR = 1.13, 95% CI 0.64 to 1.99; *p* = 0.668; I^2^ = 0%) (Figure 4). The finding was robust in the leave-one-out sensitivity analysis (Figure S5).

Visually, the funnel plot of standard error by log OR was slightly asymmetric (Figure S6). This asymmetry was imputed to two potentially missing studies on the left-side of the plot, which reduced the estimated effect size to 1.01 (95% CI 0.59 to 1.75). Egger’s linear regression confirmed the presence of publication bias for the analysis (*p* < 0.01), though Begg’s rank correlation did not.

#### 3.3.5. Musculoskeletal AEs

**Figure 5:**
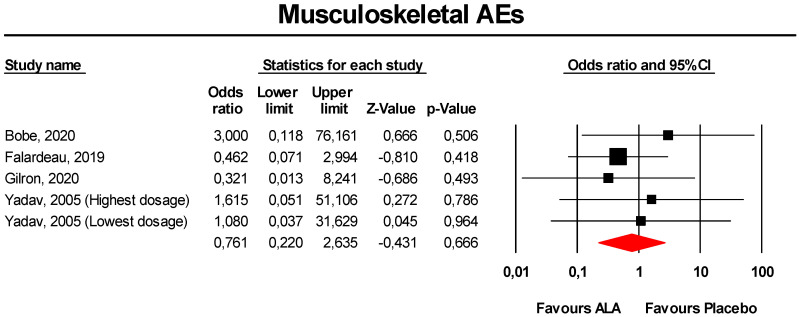
Forest plot for the risk of musculoskeletal AEs following ALA supplementation *versus* placebo.

Meta-analysis of extracted data suggested that supplementation with ALA was not associated with an increased risk of musculoskeletal AEs (OR = 0.76, 95% CI 0.22 to 2.64; *p* = 0.666; I^2^ = 0%) (Figure 5). The finding was robust in the leave-one-out sensitivity analysis (Figure S7).

Visually, the funnel plot of standard error by log OR was slightly asymmetric (Figure S8). This asymmetry was imputed to 2 potentially missing studies on the left-side of the plot, which reduced the estimated effect size to 0.50 (95% CI 0.17 to 1.51). However, neither Egger’s linear regression nor Begg’s rank correlation confirmed the presence of publication bias for the analysis (*p* > 0.05 for both tests).

#### 3.3.6. Skin AEs

**Figure 6:**
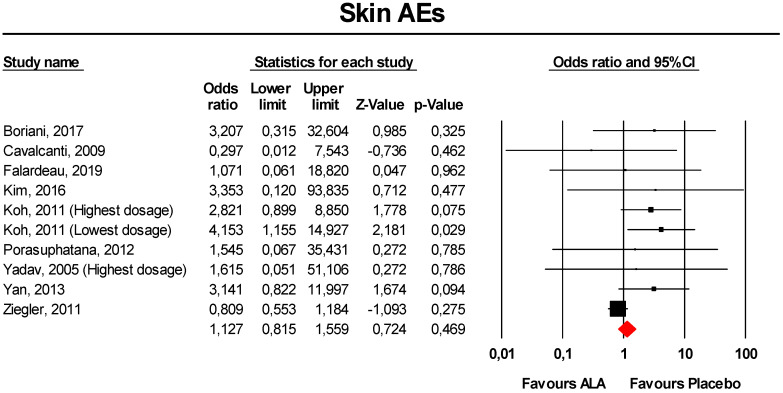
Forest plot for the risk of skin AEs following ALA supplementation *versus* placebo.

Meta-analysis of extracted data suggested that supplementation with ALA was not associated with an increased risk of skin AEs (OR = 1.13, 95% CI 0.82 to 1.56; *p* = 0.469; I^2^ = 33.6%) (Figure 6). The finding was robust in the leave-one-out sensitivity analysis (Figure S9).

Visually, the funnel plot of standard error by log OR was slightly asymmetric (Figure S10). This asymmetry was imputed to four potentially missing studies on the left-side of the plot, which reduced the estimated effect size to 0.92 (95% CI 0.68 to 1.24). However, neither Egger’s linear regression nor Begg’s rank correlation confirmed the presence of publication bias for the analysis (*p* > 0.05 for both tests).

#### 3.3.7. Infections

**Figure 7:**
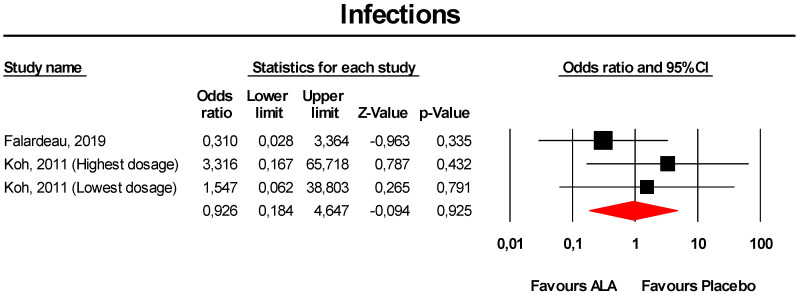
Forest plot for the risk of infections following ALA supplementation *versus* placebo.

Meta-analysis of extracted data suggested that supplementation with ALA was not associated with an increased risk of infections (OR = 0.93, 95% CI 0.18 to 4.65; *p* = 0.925; I^2^ = 0%) (Figure 7). The finding was robust in the leave-one-out sensitivity analysis (Figure S11).

Visually, the funnel plot of standard error by log OR was slightly asymmetric (Figure S12). This asymmetry was imputed to two potentially missing studies on the left-side of the plot, which reduced the estimated effect size to 0.31 (95% CI 0.08 to 1.13). However, neither Egger’s linear regression nor Begg’s rank correlation confirmed the presence of publication bias for the analysis (*p* > 0.05 for both tests).

#### 3.3.8. CV System AEs

**Figure 8:**
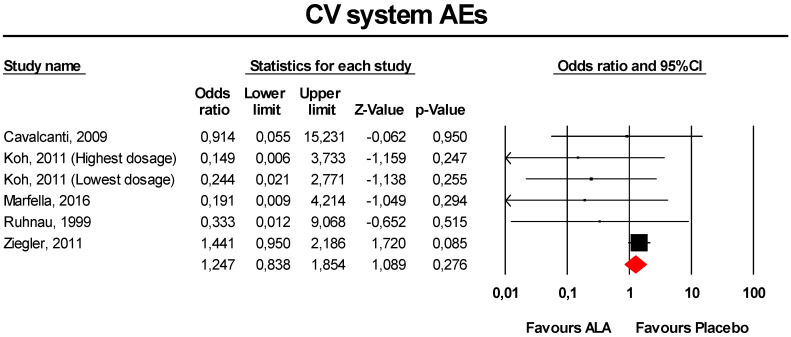
Forest plot for the risk of CV system AEs following ALA supplementation *versus* placebo.

Meta-analysis of extracted data suggested that supplementation with ALA was not associated with an increased risk of CV system AEs (OR = 1.25, 95% CI 0.84 to 1.85; *p* = 0.276; I^2^ = 15.8%) (Figure 8). The finding was robust in the leave-one-out sensitivity analysis (Figure S13).

Visually, the funnel plot of standard error by log OR was slightly asymmetric (Figure S14). This asymmetry was imputed to three potentially missing studies on the right-side of the plot, which increased the estimated effect size to 1.40 (95% CI 0.95 to 2.05). Egger’s linear regression confirmed the presence of publication bias for the analysis (*p* < 0.01), though Begg’s rank correlation did not.

#### 3.3.9. Hospitalisation

**Figure 9:**
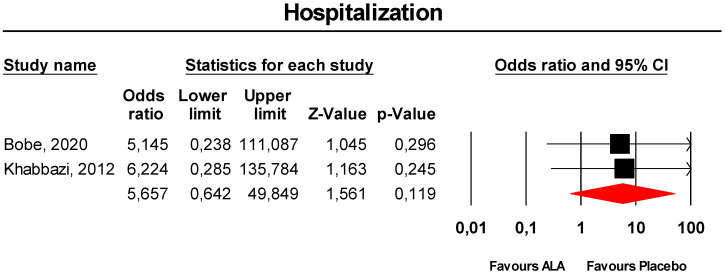
Forest plot for the risk of hospitalisation following ALA supplementation *versus* placebo.

Meta-analysis of extracted data suggested that supplementation with ALA was not associated with an increased risk of hospitalisation (OR = 5.66, 95% CI 0.64 to 49.85; *p* = 0.119; I^2^ = 0%) (Figure 9). The finding was robust in the leave-one-out sensitivity analysis (Figure S15).

#### 3.3.10. Death

**Figure 10:**
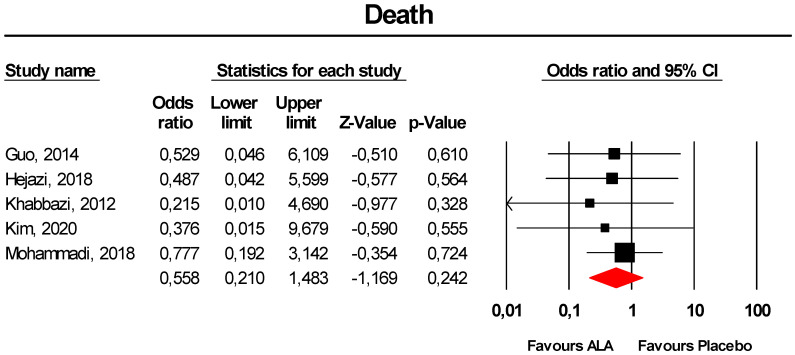
Forest plot for the risk of death following ALA supplementation *versus* placebo.

Meta-analysis of extracted data suggested that supplementation with ALA was not associated with an increased risk of death (OR = 0.56, 95% CI 0.21 to 1.48; *p* = 0.242; I^2^ = 0%) (Figure 10). The finding was robust in the leave-one-out sensitivity analysis (Figure S16).

Visually, the funnel plot of standard error by log OR was slightly asymmetric (Figure S17). This asymmetry was imputed to three potentially missing studies on the right-side of the plot, which increased the estimated effect size to 0.71 (95% CI 0.31 to 1.64). Egger’s linear regression correlation confirmed the presence of publication bias for the analysis (*p* = 0.03), though Begg’s rank correlation did not.

### 3.4. Additional Analyses

Supplementation with ALA was not associated with a significant increased risk of any AE in subsets of studies classified by smoking habit, CV disease, diabetes, pregnancy, neurological disorders, rheumatic affections, and severe renal impairment at baseline (Table 3). Furthermore, ALA supplementation was safe in children (Table 3). The findings were robust in the leave-one-out sensitivity analysis.

## 4. Discussion

In the last years, the number of individuals assuming dietary supplements has been steadily increased worldwide [90,91]. Reasons for dietary supplements’ use widely varies across the countries: in Europe, it is just limited to general health and well-being, while other countries permit use for medicinal purposes [92].

Considering that dietary supplement production and marketing are usually not strictly subjected to rigid rules as drugs are, there is a need for more data in order to confirm their safe use in the general population and frail subjects.

Pooling data from 71 randomized placebo-controlled clinical studies, this meta-analysis suggests that antioxidant supplementation with ALA was not associated with an increased risk of any treatment-emergent AE. Of note, statistical significance was not even achieved in subsets of studies categorized according to smoking habit, CV disease, presence of diabetes, pregnancy status, neurological disorders, rheumatic affections, renal impairment, and status of children/adolescent.

From a certain point of view, the current analysis strengthens findings from a large observational study considering outcomes data of 610 expectant mothers and their newborns that concluded ALA supplementation is safe in pregnancy even when administered at high doses [93].

These findings are particularly important because they encourage ALA use in a number of conditions in which ALA is actually proven to be effective. As a matter of fact, even though ALA supplementation has already been demonstrated to influence a broad spectrum of metabolic pathways including inflammation and glucose homeostasis [94,95,96], to the best of our knowledge this is the first time that ALA safety profile has been comprehensively evaluated through a pooled analysis of randomized placebo-controlled clinical studies.

Once ALA safety has been established, clinical factors for predicting treatment response should be an objective for future investigations, in order to identify the patient group that might benefit from ALA supplementation the most.

In the past, several meta-analyses showed that ALA supplementation significantly improves both positive neuropathic symptoms and neuropathic deficits to a clinically meaningful degree in diabetic patients with symptomatic polyneuropathy [97,98,99]. Furthermore, ALA was shown to promote weight loss in adults and obese children and adolescents [100,101].

Despite its strengths, this systematic review and meta-analysis has some limitations that mostly inherits from the included clinical studies. First, the effect size on the risk of hypoglycaemic episodes may be affected by variations in the underlying hypoglycaemic therapy in clinical trials enrolling diabetic patients. In fact, the well-recognized euglycaemic effect of ALA may require the adjustment of antidiabetic agents and insulin doses in patients taking antidiabetic drugs [101]. Second, gastrointestinal and CV system AEs included several nosological entities, justifying the probable presence of publication biases for the analysis. However, this limitation is strongly conditioned by the way the AEs were reported in the individual clinical trials. Indeed, most of the studies included in the meta-analysis report the cumulative incidence of gastrointestinal and CV system AEs, without regard to specific type of AEs. Third, AEs were difficult to identify when they were represented by exacerbations of the underlying disease for which ALA was tested (e.g., leg cramps in patients with peripheral polyneuropathy). Moreover, clinical trials testing different ALA regimens often reported the cumulative number of AEs for the supplementation versus placebo. As a result, a sub-analysis by ALA daily dose was not provided. Furthermore, different ALA formulations were tested across the included clinical studies. Despite this, heterogeneity was low for all assessed outcomes, proving that the results were reliable for the whole population and the considered sub-groups [102]. Finally, as per other dietary supplements, a relatively large number of studies have been carried out with open design and/or without a control group, so that they could not be included in a well-carried out meta-analysis.

Future research is needed to understand if sporadic adverse events associated with ALA use are related to the production quality of the used supplements, to other components of mixed supplements and/or to concomitant treatments or diseases, while long-term safety has been already assessed in the NATHAN (Neurological Assessment of Thioctic Acid in Diabetic Neuropathy) 1 trial [84].

## 5. Conclusions

Pooling data from the available randomized placebo-controlled clinical studies, the current meta-analysis provides data in support of the safety of the use of ALA to improve health outcomes in overall healthy individuals and in patients affected by other diseases.

## Figures and Tables

**Table 1:** Main characteristics of the clinical trials testing safety of treatment with α-lipoic acid.

Author, Year	Location	Study Design	Treatment Duration	Main Inclusion Criteria and Underlying Disease	Study Group	Enrolled Subjects (n)	Age (years; mean ± SD)	Male [n (%)]
Ahmadi, 2013 [20]	Iran	Randomized, single-blind, placebo-controlled, parallel-group, clinical study	2 months	End-stage renal disease on haemodialysis (≥2 times/week for ≥1 year)	600 mg/day α-lipoic acid	20	48.8 ± 11.2	14 (70)
					Placebo	24	48.9 ± 12.5	9 (38)
Ansar, 2011 [21]	Iran	Randomized, double-blind, placebo-controlled, parallel-group, clinical study	8 weeks	Type 2 diabetes mellitus FPG > 126 mg/dL	300 mg/day α-lipoic acid	29	49 ± 9.1	6 (21)
					Placebo	28	51.8 ± 8.3	8 (29)
Aslfalah, 2019a [22]	Iran	Randomized, double-blind, placebo-controlled, parallel-group, clinical study	8 weeks	Gestational diabetes mellitus	100 mg/day α-lipoic acid	30	30.96 ± 0.93	0 (0)
					Placebo	30	31.1 ± 0.92	0 (0)
Aslfalah, 2019b [23]	Iran	Randomized, double-blind, placebo-controlled, parallel-group, clinical study	8 weeks	Gestational diabetes mellitus	100 mg/day α-lipoic acid	30	30.96 ± 0.93	0 (0)
					Placebo	30	31.1 ± 0.92	0 (0)
Baumgartner, 2017 [24]	The Netherlands	Randomized, double-blind, placebo-controlled, crossover, clinical study	4 weeks	Impaired glucose tolerance or non-insulin-dependent type 2 diabetes BMI ≥ 20 kg/m^2^ and ≤35 kg/m^2^	600 mg/day α-lipoic acid	20	63.1 ± 5.8	16 (80)
					Placebo			
Baziar, 2020 [25]	Iran	Randomized, double-blind, placebo-controlled, parallel-group, clinical study	8 weeks	Non-insulin-dependent diabetes mellitus HbA1c < 7% BMI ≥ 18.5 kg/m^2^ and ≤29.9 kg/m^2^	1200 mg/day α-lipoic acid	35	52.66 ± 4.81	15 (43)
					Placebo	35	53.34 ± 4.45	16 (46)
Bobe, 2020 [26]	United States of America	Randomized, double-blind, placebo-controlled, parallel-group, clinical study	24 weeks	Sedentary lifestyle BMI ≥ 27 kg/m^2^ TG ≥ 150 mg/dL FPG < 125 mg/dL	600 mg/day α-lipoic acid	40	38 ± 10 *	12 (39) *
					Placebo	41	40 ± 8	16 (48) *
Boriani, 2017 [27]	Italy	Randomized, double-blind, placebo-controlled, parallel-group, clinical study	40 days	Primary tunnel carpal syndrome at least one of the following findings: anaesthesia or paraesthesia in the median nerve territory, positive Tinel sign, Phalen or reverse Phalen manoeuvres, and positive nerve conduction studies irrespective of severity	800 mg/day α-lipoic acid	32	57.3 ± 12	13 (41)
					Placebo	32	58.5 ± 11	9 (28)
Carbone, 2009 [28]	Italy	Randomized, double-blind, placebo-controlled, parallel-group, clinical study	8 weeks	Burning mouth syndrome	800 mg/day α-lipoic acid	22	NA	NA
					Placebo	22	NA	NA
Cavalcanti, 2009 [29]	Brazil	Randomized, double-blind, placebo-controlled, crossover, clinical study	30 days	Burning mouth syndrome	600 mg/day α-lipoic acid	38	63.1 (36–78) ^§^	4 (11)
					Placebo			
Durastanti, 2016 [30]	Italy	Randomized, double-blind, placebo-controlled, parallel-group, pilot clinical study	2 years	Relapsing-remitting multiple sclerosis EDSS score ≤ 3.5	800 mg/day α-lipoic acid during the first year and 400 mg/day α-lipoic acid during the second year	7	33 (26–43) °	2 (29)
					Placebo	6	28.5 (22.5–44.3) °	1 (17)
El Amrousy, 2020 [31]	Egypt	Randomized, double-blind, placebo-controlled, parallel-group, clinical study	3 months	Obese healthy children and adolescents BMI > 95th percentile for age and sex	600 mg/day α-lipoic acid	40	12.3 ± 1.5	16 (40)
					Placebo	40	12.4 ± 1.4	18 (45)
Falardeau, 2019 [32]	United States of America	Randomized, double-blind, placebo-controlled, parallel-group, clinical study	6 weeks	Unilateral acute optic neuritis	1200 mg/day α-lipoic acid	15	41.2 ± 10.51	7 (47)
					Placebo	16	36.1 ± 9.84	4 (25)
Femiano, 2002 [33]	Spain	Randomized, double-blind, placebo-controlled, parallel-group, clinical study	2 months	Burning mouth syndrome	600 mg/day α-lipoic acid	30	45 (22–68) ^§^	18 (30)
					Placebo	30		
Georgakouli, 2018 [34]	Greece	Randomized, double-blind, placebo-controlled, crossover, clinical study	4 weeks	Healthy status	600 mg/day α-lipoic acid	8	38.4 ± 5.6	8 (100)
					Placebo			
Gianturco, 2009 [35]	Italy	Randomized, double-blind, placebo-controlled, parallel-group, clinical study	4 weeks	Diabetes mellitus HbA1c < 7%	400 mg/day α-lipoic acid	7	61 ± 7	4 (57)
					Placebo	7	58 ± 16	4 (57)
Gilron, 2020 [36]	Canada	Randomized, double-blind, placebo-controlled, crossover, clinical study	5 weeks	Fibromyalgia daily moderate pain (≥4/10 on a NRS) for ≥3 months	600 mg/day α-lipoic acid during the first week; 1200 mg/day α-lipoic acid during the second week; 1800 mg/day α-lipoic acid during the third and the fourth weeks	27	57 (25–74) ^§^	5 (19)
					Placebo			
Gosselin, 2019 [37]	United States of America	Randomized, double-blind, placebo-controlled, crossover, clinical study	1 month	Sedentary lifestyle FPG ≥ 100 mg/dL and ≤125 mg/dL BMI ≥ 25 kg/m^2^ and ≤40 kg/m^2^	600 mg/day α-lipoic acid	12	47.1 ± 2.9	4 (33)
					Placebo			
Guo, 2014 [38]	United States of America	Randomized, double-blind, placebo-controlled, parallel-group, clinical study	24 weeks	Cancer patients receiving chemotherapy with cisplatin or oxaliplatin	1800 mg/day α-lipoic acid	122	55 ± 11	66 (54)
					Placebo	121	57 ± 12	63 (52)
Haghighian, 2015 [39]	Iran	Randomized, triple-blind, placebo-controlled, parallel-group, clinical study	12 weeks	Idiopathic asthenozoospermia BMI < 30 kg/m^2^	600 mg/day α-lipoic acid	24	32.98 ± 5.35 *	24 (100)
					Placebo	24	34.12 ± 4.79 *	24 (100)
Hejazi, 2018 [40]	Iran	Randomized, double-blind, placebo-controlled, parallel-group, clinical study	10 days	Candidates for enteral feeding and expected to stay in the intensive care unit for ≥7 days	2700 mg/day α-lipoic acid	40	51.2 ± 17	17 (43)
					Placebo	40	57.4 ± 19	25 (63)
Huang, 2008 [41]	United States of America	Randomized, double-blind, placebo-controlled, parallel-group, clinical study	3 months	Pubertal or postpubertal adolescents with type 1 diabetes	600–1200 mg/day (14–21 mg/kg/day) α-lipoic acid	30	14 ± 2.4	13 (43)
					Placebo	10	15 ± 1.9	7 (70)
Huerta, 2016 [42]	Spain	Randomized, double-blind, placebo-controlled, parallel-group, clinical study	10 weeks	Sedentary lifestyle BMI ≥ 27.5 kg/m^2^ and ≤40 kg/m^2^	300 mg/day α-lipoic acid	6	35.5 ± 8.4	0 (0)
					Placebo	6	41.8 ± 6.6	0 (0)
Huerta, 2015 [43]	Spain	Randomized, double-blind, placebo-controlled, parallel-group, clinical study	10 weeks	Healthy status regular menstrual cycles BMI ≥ 27.5 kg/m^2^ and ≤40 kg/m^2^	300 mg/day α-lipoic acid	26	39 ± 8 *	0 (0)
					Placebo	31	38 ± 7 *	0 (0)
Jacob, 1999 [44]	Germany	Randomized, double-blind, placebo-controlled, parallel-group, clinical study	4 weeks	Well-controlled type 2 diabetes mellitus	1800 mg/day α-lipoic acid	18	62.1 ± 3	10 (56)
					1200 mg/day α-lipoic acid	18	60.9 ± 2.2	11 (61)
					600 mg/day α-lipoic acid	19	58.1 ± 2.8	10 (53)
					Placebo	19	60.4 ± 2.4	12 (63)
Jamshidi, 2020 [45]	Iran	Randomized, double-blind, placebo-controlled, crossover, clinical study	8 weeks	β-thalassemia major	600 mg/day α-lipoic acid	20	23.5 ± 5.47	13 (65)
					Placebo			
Jariwalla, 2008 [46]	United States of America	Randomized, double-blind, placebo-controlled, parallel-group, clinical study	6 months	HIV infection HIV-RNA viral load > 10.000 copies/cm^3^ despite HAART CD4+ cell count ≥ 50 cells/mm^3^	900 mg/day α-lipoic acid	18	47.2 ± 6.8	29 (88)
					Placebo	15	43.7 ± 7.6	
Khabbazi, 2012 [47]	Iran	Randomized, double-blind, placebo-controlled, parallel-group, clinical study	8 weeks	Patients with end-stage renal disease on haemodialysis	600 mg/day α-lipoic acid	31	53.83 ± 13.29	16 (52)
					Placebo	32	54.04 ± 13.96	18 (56)
Khalili, 2017 [48]	Iran	Randomized, double-blind, placebo-controlled, parallel-group, clinical study	12 weeks	Relapsing-remitting multiple sclerosis	1200 mg/day α-lipoic acid	15	32.3 ± 6.2 *	5 (42) *
					Placebo	16	32.2 ± 10.5 *	1 (8) *
Khalili, 2014 [49]	Iran	Randomized, double-blind, placebo-controlled, parallel-group, clinical study	12 weeks	Relapsing-remitting multiple sclerosis	1200 mg/day α-lipoic acid	26	31.4 ± 6.2 *	7 (27)
					Placebo	34	28.7 ± 9 *	9 (26)
Kim, 2020 [50]	South Korea	Randomized, double-blind, placebo-controlled, parallel-group, clinical study	18 months	Geographic atrophy	1200 mg/day α-lipoic acid	26	80.6 ± 6.5	8 (31)
					Placebo	27	79 ± 7	11 (41)
Kim, 2016 [51]	South Korea	Randomized, double-blind, placebo-controlled, parallel-group, clinical study	12 weeks	Chronic schizophrenia in rehabilitation significant weight gain after starting treatment with atypical antipsychotics	600–1800 mg/day α-lipoic acid	10	40.5 ± 6.65	4 (40)
					Placebo	12	40.08 ± 9.14	7 (58)
Koh, 2011 [52]	Republic of Korea	Randomized, double-blind, placebo-controlled, parallel-group, clinical study	20 weeks	BMI ≥ 30 kg/m^2^ or BMI ≥ 27.5 kg/m^2^ and ≤40 kg/m^2^ if hypertension, diabetes mellitus and/or hypercholesterolemia coexisted	1800 mg/day α-lipoic acid	120	41.4 ± 1	82 (68)
					1200 mg/day α-lipoic acid	120	41.6 ± 1.1	79 (66)
					Placebo	120	40.7 ± 1.1	74 (62)
Lampitella, 2005 [53]	Italy	Randomized, double-blind, placebo-controlled, parallel-group, clinical study	6 months	Type 2 diabetes mellitus	600 mg/day α-lipoic acid	20	NA	NA
					Placebo	20	NA	NA
Lee, 2017 [54]	Republic of Korea	Randomized, double-blind, placebo-controlled, parallel-group, clinical study	24 weeks	Diabetic cardiac autonomic neuropathy	600-1200 mg/day α-lipoic acid	46	64.37 ± 7.8	27 (59)
					Placebo	45	62.4 ± 9.1	20 (44)
Loy, 2018 [55]	United States of America	Randomized, double-blind, placebo-controlled, parallel-group, pilot clinical study	2 years	Multiple sclerosis disability progression in absence of clinical relapse for 5 years EDSS ≤ 6.0 ability to walk ≥ 25 feet without aid	1200 mg/day α-lipoic acid	11	55.8 ± 5.7	5 (45)
					Placebo	10	55.7 ± 4.1	5 (50)
López-D’alessandro, 2011 [56]	Argentina	Randomized, double-blind, placebo-controlled, parallel-group, clinical study	2 months	Burning mouth syndrome	600 g/day α-lipoic acid	20	NA	NA
					Placebo	60	NA	NA
López-Jornet, 2009 [57]	Spain	Randomized, double-blind, placebo-controlled, parallel-group, clinical study	8 weeks	Burning mouth syndrome	800 mg/day α-lipoic acid	30	64.37 ± 11.61	6 (10)
					Placebo	30		
Magis, 2007 [58]	Belgium	Randomized, double-blind, placebo-controlled, parallel-group, clinical study	3 months	Migraine with or without aura	600 mg/day α-lipoic acid	26	37.46 ± 13.43	4 (15)
					Placebo	18	38.94 ± 8.05	2 (11)
Manning, 2013 [59]	New Zeland	Randomized, double-blind, placebo-controlled, parallel-group, clinical study	1 year	Metabolic syndrome	600 mg/day α-lipoic acid	34	55 ± 10	14 (41)
					Placebo	40	57 ± 9	15 (38)
Marfella, 2016 [60]	Italy	Randomized, double-blind, placebo-controlled, parallel-group, clinical study	12 months	Takotsubo cadiomyopathy	600 mg/day α-lipoic acid	24	63.7 ± 6.5	0 (0)
					Placebo	24	63.9 ± 5.2	0 (0)
Marshall, 1982 [61]	United Kingdom	Randomized, double-blind, placebo-controlled, parallel-group, clinical study	24 weeks	Alcohol related liver disease	300 mg/day α-lipoic acid	20	50.7 ± 1.9	17 (85)
					Placebo	20	46.4 ± 2.7	15 (75)
Martins, 2009 [62]	Brazil	Randomized, double-blind, placebo-controlled, parallel-group, clinical study	3 months	Sickle cell disease	200 mg/day α-lipoic acid	10	17.7 ± 9.6	6 (60)
					Placebo	10	17 ± 11	5 (50)
				Sickle cell trait	200 mg/day α-lipoic acid	10	31.3 ± 15.4	2 (20)
					Placebo	10	29.7 ± 10.8	2 (20)
				Healthy status	200 mg/day α-lipoic acid	10	23.5 ± 11	4 (40)
					Placebo	10	23.3 ± 11	3 (30)
Mendes, 2014 [63]	Brazil	Randomized, double-blind, placebo-controlled, parallel-group, clinical study	12 weeks	Arterial hypertension	600 mg/day α-lipoic acid	32	NA	NA
					Placebo	28	NA	NA
Mendoza-Núñez, 2019 [64]	Mexico	Randomized, double-blind, placebo-controlled, parallel-group, clinical study	6 months	Type 2 diabetes mellitus without complications or comorbidity, treated with two tablets of glibenclamide/metformin (5/500 mg) per day BMI < 35 kg/m^2^ sedentary lifestyle	600 mg/day α-lipoic acid	50	63 ± 1 *	NA
					Placebo	50	64 ± 1 *	NA
Mirtaheri, 2014 [65]	Iran	Randomized, double-blind, placebo-controlled, parallel-group, clinical study	8 weeks	Rheumatoid arthritis	1200 mg/day α-lipoic acid	35	36.09 ± 8.77 *	0 (0)
					Placebo	35	38.28 ± 8.63 *	0 (0)
Mohammadi, 2018 [66]	Iran	Randomized, double-blind, placebo-controlled, parallel-group, clinical study	12 weeks	Previous thrombotic or embolic stroke BMI ≥ 18.5 kg/m^2^ and ≤35 kg/m^2^	600 mg/day α-lipoic acid	40	62.33 ± 6.19	NA
					Placebo	40	64.23 ± 8.01	NA
Mohammadi, 2015 [67]	Iran	Randomized, double-blind, placebo-controlled, parallel-group, clinical study	12 weeks	Spinal cord injury since ≥ 1 year BMI ≥ 18.5 kg/m^2^	600 mg/day α-lipoic acid	28	39 ± 6.44	28 (100)
					Placebo	30	36.8 ± 7.48	30 (100)
Mollo, 2012 [68]	Italy	Randomized, double-blind, placebo-controlled, parallel-group, clinical study	5 weeks	Type 1 diabetes	600 mg/day α-lipoic acid	26	43 ± 9	15 (58)
					Placebo	25	46 ± 11	12 (48)
Monroy Guízar, 2018 [69]	Mexico	Randomized, double-blind, placebo-controlled, parallel-group, clinical study	3 months	Idiopathic carpal tunnel syndrome	600 mg/day α-lipoic acid	10	45.3 ^†^	1 (10)
					Placebo	10	48.4 ^†^	1 (10)
Palacios-Sánchez, 2015 [70]	Spain	Randomized, double-blind, placebo-controlled, parallel-group, clinical study	2 months	Burning mouth syndrome	600 mg/day α-lipoic acid	30	62.13 (36–86) ^§^	5 (8)
					Placebo	30		
Porasuphatana, 2012 [71]	Thailand	Randomized, double-blind, placebo-controlled, parallel-group, clinical study	6 months	Type 2 diabetes mellitus with microalbuminuria	1200 mg/day α-lipoic acid	7	47.07 ± 2.18	1 (14)
					900 mg/day α-lipoic acid	7	44 ± 2	1 (14)
					600 mg/day α-lipoic acid	8	45.7 ± 1.68	3 (38)
					300 mg/day α-lipoic acid	8	42.5 ± 1.12	4 (50)
					Placebo	8	42.9 ± 2.52	1 (13)
Pourghasem Gargari, 2014 [72]	Iran	Randomized, double-blind, placebo-controlled, parallel-group, clinical study	8 weeks	Rheumatoid arthritis DAS28 < 5.1 BMI < 40 kg/m^2^	1200 mg/day α-lipoic acid	35	36.1 ± 8.8	0 (0)
					Placebo	35	38.3 ± 8.6	0 (0)
Rahmanabadi, 2019 [4]	Iran	Randomized, double-blind, placebo-controlled, parallel-group, clinical study	12 weeks	Non-alcoholic fatty liver disease BMI ≥ 30 kg/m^2^ and ≤40 kg/m^2^	1200 mg/day α-lipoic acid	25	40.28 ± 5.5	13 (52)
					Placebo	25	37.52 ± 9.67	14 (56)
Ruhnau, 1999 [73]	Germany	Randomized, double-blind, placebo-controlled, parallel-group, clinical study	3 weeks	Type 2 diabetes mellitus with distal symmetrical polyneuropathy	1800 mg/day α-lipoic acid	12	60.5 ± 6.9	6 (50)
					Placebo	12	62.1 ± 4.5	6 (50)
Safa, 2014 [74]	Iran	Randomized, double-blind, placebo-controlled, parallel-group, clinical study	12 months	End-stage renal disease on haemodialysis ≥ 6 months	600 mg/day α-lipoic acid	30	59.3 ± 10.47	21 (70)
					Placebo	31	55.2 ± 13.43	21 (68)
Sammour, 2019 [75]	Egypt	Randomized, triple-blind, placebo-controlled, parallel-group, clinical study	6 weeks	Primary caesarean section in singleton term pregnancy	1200 mg/day α-lipoic acid	51	25.3 ± 5.1	0 (0)
					Placebo	51	25.1 ± 5.4	0 (0)
Sardu, 2017 [76]	Italy	Randomized, double-blind, placebo-controlled, parallel-group, clinical study	12 months	Paroxysmal, symptomatic atrial fibrillation ≥ 6 months refractory to ≥1 class 1–3 antiarrhythmic drugs and treated with catheter ablation	600 mg/day α-lipoic acid	33	58.8 ± 6.7	15 (45)
					Placebo	40	61.5 ± 8.1	23 (58)
Scaramuzza, 2015 [77]	Italy	Randomized, double-blind, placebo-controlled, parallel-group, pilot clinical study	6 months	Type 1 diabetes endothelial dysfunction	800 mg/day α-lipoic acid	25	16.1 ± 3.1	15 (60)
					Placebo	27	16 ± 3.4	16 (59)
Sola, 2005 [78]	United Stated of America	Randomized, double-blind, placebo-controlled, parallel-group, clinical study	4 weeks	Metabolic syndrome	300 mg/day α-lipoic acid	15	46 ± 15	5 (33)
					Placebo	14	44 ± 13	6 (43)
Spain, 2017 [79]	United Stated of America	Randomized, double-blind, placebo-controlled, parallel-group, clinical study	2 years	Multiple sclerosis disability progression in absence of clinical relapse for 5 years	1200 mg/day α-lipoic acid	27	57.9 ± 6.7	11 (41)
					Placebo	24	59.7 ± 6	9 (38)
Sun, 2012 [80]	China	Randomized, blind, placebo-controlled, parallel-group, clinical study	3 months	Dry form of age-related macular degeneration	600 mg/day α-lipoic acid	32	65.8 ± 7.9	11 (35)
					Placebo	30	64.5 ± 8.1	10 (33)
Tromba, 2019 [81]	Italy	Randomized, double-blind, placebo-controlled, parallel-group, clinical study	12 weeks	BMI ≥ 85th percentile for age and sex	800 mg/day α-lipoic acid	34	11.5 ± 1.9 *	16 (50) *
					Placebo	33	11.1 ± 2.1 *	20 (63) *
Udupa, 2013 [82]	India	Randomized, double-blind, placebo-controlled, parallel-group, clinical study	90 days	Type 2 diabetes mellitus FGP ≥ 110 mg/dL and ≤250 mg/dL	300 mg/day α-lipoic acid	25	53.5 ± 1.4	12 (48)
					Placebo	25	53.8 ± 2.1	15 (60)
Vincent, 2007 [83]	United States of America	Randomized, double-blind, placebo-controlled, parallel-group, clinical study	3 months	ABI ≥ 0.3 and ≤0.9 claudication pain with walking	600 mg/day α-lipoic acid	16	75.1 ± 8.2	9 (56)
					Placebo	12	70.7 ± 18.9	6 (50)
Yadav, 2005 [84]	United States of America	Randomized, double-blind, placebo-controlled, parallel-group, pilot clinical study	14 days	Multiple sclerosis EDSS score ≤ 7.5	2400 mg/day α-lipoic acid	8	44.5 (34–56) ^§^	0 (0)
					1200 mg/day α-lipoic acid	16	NA	2 (13)
					Placebo	9	50 (36–66) ^§^	2 (22)
Yan, 2013 [85]	China	Randomized, double-blind, placebo-controlled, crossover, clinical study	8 weeks	BMI ≥ 25 kg/m^2^ ≥1 of borderline hypertension, dyslipidemia, or impaired FPG	1200 mg/day α-lipoic acid	103	NA	NA
					Placebo			
Zembron-Lacny, 2013 [86]	Poland	Randomized, double-blind, placebo-controlled, crossover, clinical study	10 days	Healthy status	1200 mg/day α-lipoic acid	16	20.7 ± 0.9	16 (100)
					Placebo			
Zembron-Lacny, 2009 [87]	Poland	Randomized, double-blind, placebo-controlled, crossover, clinical study	8 days	Physical education students healthy status forced training experience ≥3 years	1200 mg/day α-lipoic acid	13	25.5 ± 6	13 (100)
					Placebo			
Ziegler, 2011 [88]	Canada, Croatia, Denmark, France, Italy, Spain, The Netherlands, United Kingdom, United States of America	Randomized, double-blind, placebo-controlled, parallel-group, clinical study	4 years	Type 1 or 2 diabetes (duration ≥1 year) stage 1 or 2a distal symmetric sensorimotor polyneuropathy due to diabetes stable insulin regimen NIS_[LL]_+7 ≥ 2 one of the following abnormalities: abnormal nerve conduction attributes in two separate nerves ≥ 99th percentile for distal latency or ≤1st percentile for nerve conduction velocity or amplitude OR HRBD ≥ 1st percentile or TSS in the feet< 5	600 mg/day α-lipoic acid	231	53.3 ± 8.3	152 (66)
					Placebo	225	53.9 ± 7.6	154 (67)
Ziegler, 2006 [89]	Israel and Russia	Randomized, double-blind, placebo-controlled, parallel-group, clinical study	5 weeks	Type 1 or 2 diabetes HbA1c < 10% symptomatic distal symmetric polyneuropathy due to diabetes TSS > 7.5 NIS_[LL]_ ≥ 2 absent or decreased pain sensation according to pin-prick test	1800 mg/day α-lipoic acid	46	59 ± 9	19 (41)
					1200 mg/day α-lipoic acid	47	59 ± 12	19 (40)
					600 mg/day α-lipoic acid	45	56 ± 12	20 (44)
					Placebo	43	57 ± 11	15 (35)

* data refer to safety population; ^§^ data reported as median (variation range); ° data reported as median (interquartile range); ^†^ data reported as mean; ABI = Ankle brachial index; BMI = Body mass index; CVD = Cardiovascular disease; DAS28 = Disease activity score in 28 joints; EDSS = Expanded disability status scale; HIV = Human immunodeficiency virus; HRBD = Heart rate during deep breathing; NA = Not available; NIS_[LL]_ = Neuropathy impairment score — subscore for lower limbs; NIS_[LL]_+7 = Neuropathy impairment score—subscore for lower limbs and seven nerve conduction tests score; NRS = Numerical rating scale; FPG = Fasting plasma glucose; TSS = Total symptom score.

**Table 2:** Quality of bias assessment of the included studies according to Cochrane guidelines.

Author, Year	Sequence Generation	Allocation Concealment	Blinding to Participants, Personnel and Outcome Assessment	Incomplete Outcome Data	Selective Outcome Reporting	Other Potential Threats to Validity
Ahmadi, 2013 [20]	L	L	H	L	L	U
Ansar, 2011 [21]	L	L	L	L	U	L
Aslfalah, 2019a [22]	L	L	L	L	L	L
Aslfalah, 2019b [23]	L	L	L	L	L	L
Baumgartner, 2017 [24]	L	L	L	L	L	L
Baziar, 2020 [25]	L	L	L	L	L	L
Bobe, 2020 [26]	L	L	L	L	L	L
Boriani, 2017 [27]	L	L	L	L	L	L
Carbone, 2009 [28]	L	L	L	L	L	L
Cavalcanti, 2009 [29]	L	L	L	L	L	L
Durastanti, 2016 [30]	L	L	L	U	U	U
El Amrousy, 2020 [31]	L	L	L	L	L	L
Falardeau, 2019 [32]	L	L	L	L	L	L
Femiano, 2002 [33]	U	L	L	L	U	U
Georgakouli, 2018 [34]	L	L	L	L	L	L
Gianturco, 2009 [35]	L	L	L	L	U	L
Gilron, 2020 [36]	L	L	L	L	L	L
Gosselin, 2019 [37]	L	L	L	L	L	L
Guo, 2014 [38]	L	L	L	L	L	L
Haghighian, 2015 [39]	L	L	L	L	L	L
Hejazi, 2018 [40]	L	L	L	L	L	L
Huang, 2008 [41]	L	L	L	L	L	L
Huerta, 2016 [42]	L	L	L	L	L	L
Huerta, 2015 [43]	L	L	L	L	L	L
Jacob, 1999 [44]	L	L	L	L	U	H
Jamshidi, 2020 [45]	L	L	L	L	L	L
Jariwalla, 2008 [46]	L	L	L	L	U	H
Khabbazi, 2012 [47]	L	L	L	L	L	L
Khalili, 2017 [48]	L	L	L	L	L	L
Khalili, 2014 [49]	L	L	L	L	L	L
Kim, 2020 [50]	L	L	L	L	L	L
Kim, 2016 [51]	L	L	L	L	L	L
Koh, 2011 [52]	L	L	L	L	L	L
Lampitella, 2005 [53]	L	U	U	L	L	U
Lee, 2017 [54]	L	L	L	L	L	L
Loy, 2018 [55]	L	L	L	L	L	L
López- D’Alessandro, 2011 [56]	L	L	L	H	H	U
López-Jornet, 2009 [57]	L	L	L	L	L	L
Magis, 2007 [58]	L	L	L	L	L	L
Manning, 2013 [59]	L	L	L	L	L	L
Marfella, 2016 [60]	L	L	U	L	L	U
Marshall, 1982 [61]	L	L	L	L	L	L
Martins, 2009 [62]	L	L	U	L	L	U
Mendes, 2014 [63]	L	L	L	L	H	U
Mendoza- Núñez, 2019 [64]	L	L	L	L	L	L
Mirtaheri, 2014 [65]	L	L	L	L	L	L
Mohammadi, 2018 [66]	L	L	L	L	L	L
Mohammadi, 2015 [67]	L	L	L	L	L	L
Mollo, 2012 [68]	L	L	L	L	L	L
Monroy Guízar, 2018 [69]	L	L	L	L	L	L
Palacios- Sánchez, 2015 [70]	L	L	L	L	L	L
Porasuphatana, 2012 [71]	L	L	L	L	L	H
Pourghasem Gargari, 2014 [72]	L	L	L	L	L	L
Rahmanabadi, 2019 [4]	L	L	L	L	L	L
Ruhnau, 1999 [73]	L	L	L	L	L	L
Safa, 2014 [74]	L	L	L	L	L	L
Sammour, 2019 [75]	L	L	L	L	L	L
Sardu, 2017 [76]	L	L	L	L	L	L
Scaramuzza, 2015 [77]	L	L	L	L	L	L
Sola, 2005 [78]	L	L	L	L	L	L
Spain, 2017 [79]	L	L	L	L	L	L
Sun, 2012 [80]	L	U	U	L	L	U
Tromba, 2019 [81]	L	L	L	L	L	L
Udupa, 2013 [82]	L	L	L	L	L	L
Vincent, 2007 [83]	L	L	L	L	L	L
Yadav, 2005 [84]	L	L	L	L	L	L
Yan, 2013 [85]	L	L	L	L	L	L
Zembron- Lacny, 2013 [86]	L	L	L	L	L	L
Zembron- Lacny, 2009 [87]	L	L	L	L	L	L
Ziegler, 2011 [88]	L	L	L	L	L	L
Ziegler, 2006 [89]	L	L	L	L	L	L

H = High risk of bias; L = Low risk of bias; U = Unclear risk of bias.

**Table 3:** Subgroup analyses for the risk of treatment-emergent AEs, stratified by smoking habit, cardiovascular disease, presence of diabetes, pregnancy, neurological disorders, rheumatic affections, age, and severe renal impairment at baseline.

AEs		Smoking Habit		Cardiovascular Disease		Diabetes		Pregnancy		Neurological Disorders		Rheumatic Affections		Children and/or Adolescents		Severe Renal Impairment	
		Yes	No	Yes	No	Yes	No	Yes	No	Yes	No	Yes	No	Yes	No	Yes	No
Gastrointestinal AEs	Number of reported AEs (active arm/placebo arm)	-/-	4/2	2/0	97/88	137/77	17/14	3/2	180/97	144/76	-/-	5/2	4/3	3/2	180/97	-/-	94/81
	Odd ratio	-	1.192	2.734	1.103	1.267	1.155	1.531	1.313	1.295	-	2.841	1.433	1.705	1.309	-	1.158
	95% CI (lower limit; upper limit)	-	0.265; 5.361	0.273; 27.383	0.781; 1.558	0.879; 1.827	0.540; 2.468	0.245; 9.574	0.966; 1.784	0.897; 1.869	-	0.500; 16.138	0.300; 6.833	0.260; 11.156	0.964; 1.779	-	0.811; 1.653
	Z-value	-	0.229	0.856	0.556	1.268	0.371	0.456	1.740	1.382	-	1.178	0.451	0.556	1.724	-	0.809
	I^2^ (%)	-	0	0	0	50	0	0	0	48	-	0	0	0	0	-	0
	*P*-value	-	0.819	0.392	0.578	0.205	0.711	0.649	0.082	0.167	-	0.239	0.652	0.578	0.085	-	0.418
Neurological AEs	Number of reported AEs (active arm/placebo arm)	-/-	6/2	1/0	19/18	10/0	18/14	-/-	50/23	25/9	-/-	8/6	0/1	-/-	50/23	-/-	22/16
	Odd ratio	-	1.024	3.078	1.153	2.368	1.268	-	1.526	1.718	-	1.474	0.315	-	1.526	-	3.078
	95% CI (lower limit; upper limit)	-	0.236; 4.442	0.122; 77.905	0.544; 2.442	0.884; 2.634	0.552; 2.914	-	0.884; 2.634	0.742; 3.977	-	0.432; 5.027	0.012; 7.999	-	0.884; 2.634	-	0.122; 77.905
	Z-value	-	0.032	0.682	0.371	1.517	0.560	-	1.517	1.264	-	0.619	−0.700	-	1.517	-	0.682
	I^2^ (%)	-	0	0	0	0	0	-	0	0	-	0	0	-	0	-	0
	*P*-value	-	0.974	0.495	0.711	0.129	0.575	-	0.129	0.206	-	0.536	0.484	-	0.129	-	0.495
Psychiatric AEs	Number of reported AEs (active arm/placebo arm)	-/-	2/0	-/-	30/25	26/25	4/0	-/-	30/25	26/25	-/-	-/-	2/0	-/-	30/25	-/-	28/25
	Odd ratio	-	5.145	-	1.131	1.014	5.071	-	1.131	1.014	-	-	5.145	-	1.131	-	1.073
	95% CI (lower limit; upper limit)	-	0.238; 111.087	-	0.644; 1.986	0.566; 1.817	0.582; 44.174	-	0.644; 1.986	0.566; 1.817	-	-	0.238; 111.087	-	0.644; 1.986	-	0.605; 1.903
	Z-value	-	1.045	-	0.429	0.048	1.470	-	0.429	0.048	-	-	1.045	-	0.429	-	0.242
	I^2^ (%)	-	0	-	0	0	0	-	0	0	-	-	0	-	0	-	0
	*P*-value	-	0.296	-	0.668	0.962	0.142	-	0.668	0.962	-	-	0.296	-	0.668	-	0.809
Musculoskeletal AEs	Number of reported AEs (active arm/placebo arm)	-/-	1/0	-/-	3/5	-/-	3/4	-/-	5/5	4/4	-/-	0/1	1/0	-/-	5/5	-/-	3/5
	Odd ratio	-	3.000	-	0.625	-	0.738	-	0.761	0.683	-	0.321	3.000	-	0.761	-	0.625
	95% CI (lower limit; upper limit)	-	0.118; 76.161	-	0.147; 2.661	-	0.146; 3.723	-	0.220; 2.635	0.156; 2.997	-	0.013; 8.241	0.118; 76.161	-	0.220; 2.635	-	0.147; 2.661
	Z-value	-	0.666	-	−0.636	-	−0.368	-	−0.431	−0.505	-	−0.686	0.666	-	−0.431	-	−0.636
	I^2^ (%)	-	0	-	0	-	0	-	0	0	-	0	0	-	0	-	0
	*P*-value	-	0.506	-	0.525	-	0.713	-	0.666	0.614	-	0.493	0.506	-	0.666	-	0.525
Skin AEs	Number of reported AEs (active arm/placebo arm)	-/-	21/4	-/-	92/94	83/90	14/6	-/-	139/103	83/91	1/0	-/-	-/-	-/-	139/103	2/0	104/95
	Odd ratio	-	2.821	-	0.912	0.816	2.258	-	1.127	0.819	3.353	-	-	-	1.127	1.545	0.932
	95% CI (lower limit; upper limit)	-	0.899; 8.850	-	0.635; 1.308	0.559; 1.191	0.851; 5.992	-	0.815; 1.559	0.563; 1.192	0.120; 93.835	-	-	-	0.815; 1.559	0.067; 35.431	0.653; 1.331
	Z-value	-	1.778	-	−0.502	−1.052	1.636	-	0.724	−1.041	0.712	-	-	-	0.724	0.272	−0.387
	I^2^ (%)	-	0	-	29	0	0	-	34	0	0	-	-	-	34	0	36
	*P*-value	-	0.075	-	0.616	0.293	0.102	-	0.469	0.298	0.477	-	-	-	0.469	0.785	0.699
Infections	Number of reported AEs (active arm/placebo arm)	-/-	3/0	-/-	1/3	-/-	1/3	-/-	5/3	1/3	-/-	-/-	-/-	-/-	5/3	-/-	4/3
	Odd ratio	-	3.316	-	0.310	-	0.310	-	0.926	0.310	-	-	-	-	0.926	-	0.780
	95% CI (lower limit; upper limit)	-	0.167; 65.718	-	0.028; 3.364	-	0.028; 3.364	-	0.184; 4.647	0.028; 3.364	-	-	-	-	0.184; 4.647	-	0.121; 5.028
	Z-value	-	0.787	-	−0.963	-	−0.963	-	−0.094	−0.963	-	-	-	-	−0.094	-	−0.262
	I^2^ (%)	-	0	-	0	-	0	-	0	0	-	-	-	-	0	-	32
	*P*-value	-	0.432	-	0.335	-	0.335	-	0.925	0.335	-	-	-	-	0.925	-	0.793
CV system AEs	Number of reported AEs (active arm/placebo arm)	-/-	0/1	0/2	71/53	71/54	1/3	-/-	73/60	71/54	-/-	-/-	0/1	-/-	73/60	-/-	71/57
	Odd ratio	-	0.149	0.191	1.441	1.409	0.450	-	1.247	1.409	-	-	0.333	-	1.247	-	1.313
	95% CI (lower limit; upper limit)	-	0.006; 3.733	0.009; 4.214	0.950; 2.186	0.932; 2.130	0.056; 3.608	-	0.838; 1.854	0.932; 2.130	-	-	0.012; 9.068	-	0.838; 1.854	-	0.875; 1.972
	Z-value	-	−1.159	−1.049	1.720	1.625	−0.752	-	1.089	1.625	-	-	−0.652	-	1.089	-	1.314
	I^2^ (%)	-	0	0	0	0	0	-	16	0	-	-	0	-	16	-	27
	*P*-value	-	0.247	0.294	0.085	0.104	0.452	-	0.276	0.104	-	-	0.515	-	0.276	-	0.189
Hospitalisation	Number of reported AEs (active arm/placebo arm)	-/-	4/0	-/-	2/0	-/-	2/0	-/-	4/0	-/-	-/-	-/-	2/0	-/-	4/0	2/0	2/0
	Odd ratio	-	5.657	-	5.145	-	5.145	-	5.657	-	-	-	5.145	-	5.657	6.224	5.145
	95% CI (lower limit; upper limit)	-	0.642; 49.849	-	0.238; 111.087	-	0.238; 111.087	-	0.642; 49.849	-	-	-	0.238; 111.087	-	0.642; 49.849	0.285; 135.784	0.238; 111.087
	Z-value	-	1.561	-	1.045	-	1.045	-	1.561	-	-	-	1.045	-	1.561	1.163	1.045
	I^2^ (%)	-	0	-	0	-	0	-	0	-	-	-	0	-	0	0	0
	*P*-value	-	0.119	-	0.296	-	0.296	-	0.119	-	-	-	0.296	-	0.119	0.245	0.296
Death	Number of reported AEs (active arm/placebo arm)	-/-	0/2	4/5	-/-	-/-	1/2	-/-	6/12	1/3	-/-	-/-	-/-	-/-	6/12	0/2	6/9
	Odd ratio	-	0.215	0.777	-	-	0.529	-	0.558	0.468	-	-	-	-	0.558	0.215	0.657
	95% CI (lower limit; upper limit)	-	0.010; 4.690	0.192; 3.142	-	-	0.046; 6.109	-	0.210; 1.483	0.066; 3.300	-	-	-	-	0.210; 1.483	0.010; 4.690	0.222; 1.947
	Z-value	-	−0.977	−0.354	-	-	−0.510	-	−1.169	−0.762	-	-	-	-	−1.169	−0.977	−0.758
	I^2^ (%)	-	0	0	-	-	0	-	0	0	-	-	-	-	0	0	0
	*P*-value	-	0.328	0.724	-	-	0.610	-	0.242	0.446	-	-	-	-	0.242	0.328	0.448

AEs = Adverse events; CI = Confidence Intervals.

## Supplementary Materials

The following are available online at https://www.mdpi.com/2076-3921/9/10/1011/s1, Figure S1: Plots showing leave-one-out sensitivity analysis for the risk of gastrointestinal AEs following ALA supplementation versus placebo, Figure S2: Funnel plot detailing publication bias for the risk of gastrointestinal AEs following ALA supplementation versus placebo, Figure S3: Plot showing leave-one-out sensitivity analysis for the risk of neurological AEs following ALA supplementation versus placebo, Figure S4: Funnel plot detailing publication bias for the risk of neurological AEs following ALA supplementation versus placebo, Figure S5: Plot showing leave-one-out sensitivity analysis for the risk of psychiatric disorders following ALA supplementation versus placebo, Figure S6: Plot showing leave-one-out sensitivity analysis for the risk of musculoskeletal AEs following ALA supplementation versus placebo, Figure S7: Funnel plot detailing publication bias for the risk of musculoskeletal AEs following ALA supplementation versus placebo, Figure S8: Plot showing leave-one-out sensitivity analysis for the risk of skin AEs following ALA supplementation versus placebo, Figure S9: Funnel plot detailing publication bias for the risk of skin AEs following ALA supplementation versus placebo, Figure S10: Plot showing leave-one-out sensitivity analysis for the risk of infections following ALA supplementation versus placebo, Figure S11: Funnel plot detailing publication bias for the risk of infections following ALA supplementation versus placebo, Figure S12: Plot showing leave-one-out sensitivity analysis for the risk of CV system AEs following ALA supplementation versus placebo, Figure S13: Funnel plot detailing publication bias for the risk of CV system AEs following ALA supplementation versus placebo, Figure S14: Plot showing leave-one-out sensitivity analysis for the risk of hospitalisation following ALA supplementation versus placebo, Figure S15: Plot showing leave-one-out sensitivity analysis for the risk of death following ALA supplementation versus placebo, Figure S16: Funnel plot detailing publication bias for the risk of death following ALA supplementation versus placebo, File A: PRISMA Checklist, File B: Studies excluded from the systematic review after assessment.
