# Supplementary material for: Safety Evaluation of α-Lipoic Acid Supplementation: A Systematic Review and Meta-Analysis of Randomized Placebo-Controlled Clinical Studies
Source: Antioxidants (Basel). 2020 Oct 19;9(10):1011. doi: 10.3390/antiox9101011 (PMC7603186; doi:10.3390/antiox9101011)
Supplement: Supplementary file 1 [file antioxidants-09-01011-s001.zip › Appendix B_14092020.doc]

**Appendix B *-*** Studies excluded from the systematic review after assessment.

- **Pre-print papers (n= 2)**
  1. Kaviyani N, Keshavarz SA, Abbasi B. Effect of Alpha-lipoic Acid Supplementation on Glycemic Control in the Patients with Metabolic Syndrome: A Randomized Clinical Trial. Research Square; 2020. doi: 10.21203/rs.3.rs-37045/v1. Available at: <https://europepmc.org/article/ppr/ppr185357> [accessed 5 August 2020]. [Preprint]
  2. Mohammadshahi M, Zakizadeh E, Ahmadi Angali K, Ravanbakhsh M, Helli B. The effect of alpha-lipoic acid supplementation and Electrical isotonic contraction on anthropometric measurements, body composition, angiogenesis factors, sirtunin-1, and PGC1-α in obese people undergoing a weight loss diet. Available at: <https://europepmc.org/article/med/32580620> [accessed 30 July 2020]. [Preprint]
- **Study protocols (n= 6)**
  1. Cave AE, Chang DH, Münch GW, Steiner GZ. Efficacy of Cognition Support Formula® on cognitive function in older adults with subjective cognitive impairment: a protocol for a 26-week, randomised, double-blind, placebo-controlled trial. Trials. 2019 Jun 10;20(1):345. doi: 10.1186/s13063-019-3431-3.
  2. Gilron I, Tu D, Holden R, Towheed T, Vandenkerkhof E, Milev R. Combination Analgesic Development for Enhanced Clinical Efficacy (CADENCE Trial): Study Protocol for a Double-Blind, Randomized, Placebo-Controlled Crossover Trial of an Alpha-Lipoic Acid - Pregabalin Combination for the Treatment of Fibromyalgia Pain. JMIR Res Protoc. 2017 Aug 4;6(8):e154. doi: 10.2196/resprot.8001.
  3. Gilron I, Tu D, Holden R, Jackson AC, Ghasemlou N, Duggan S, Vandenkerkhof E, Milev R. Pain Improvement With Novel Combination Analgesic Regimens (PAIN-CARE): Randomized Controlled Trial Protocol. JMIR Res Protoc. 2017 Jun 8;6(6):e111. doi: 10.2196/resprot.7493.
  4. Gilron I, Tu D, Holden R, Towheed T, Ziegler D, Wang L, Milev R, Gray C. Innovations in the Management of Musculoskeletal Pain With Alpha-Lipoic Acid (IMPALA Trial): Study protocol for a Double-Blind, Randomized, Placebo-Controlled Crossover Trial of Alpha-Lipoic Acid for the Treatment of Fibromyalgia Pain. JMIR Res Protoc. 2017 Mar 28;6(3):e41. doi: 10.2196/resprot.7198.
  5. Mohammadshahi M, Zakizadeh E, Ahmadi-Angali K, Ravanbakhsh M, Helli B. The effect of alpha-lipoic acid supplementation and electrical isotonic contraction on anthropometric parameters, body composition and angiogenesis factor, sirtunin-1 and peroxisome proliferator-activated receptor-γ coactivator-1α in obese people under a weight loss regime: A study protocol for a randomized controlled clinical trial. Nutr Health. 2020 Jun 25:260106020926838. doi: 10.1177/0260106020926838.
  6. Navarese EP, Mollo R, Buffon A. Effect of alpha lipoic acid on cardiac autonomic dysfunction and platelet reactivity in type 1 diabetes: rationale and design of the AUTOnomic function and platelet REACTivity trial (AUTO-REACT protocol). Diabetes Res Clin Pract. 2011 Jun;92(3):375-9. doi: 10.1016/j.diabres.2010.09.003.
- **Lack of an appropriate placebo-controlled design for the supplementation (n= 64)**
  1. Ali AM, Awad TG, Al-Adl NM. Efficacy of combined topiramate/thioctic acid therapy in migraine prophylaxis. Saudi Pharm J. 2010 Oct;18(4):239-43. doi: 10.1016/j.jsps.2010.07.006.
  2. Alleva R, Tomasetti M, Sartini D, Emanuelli M, Nasole E, Di Donato F, Borghi B, Santarelli L, Neuzil J. alpha-Lipoic acid modulates extracellular matrix and angiogenesis gene expression in non-healing wounds treated with hyperbaric oxygen therapy. Mol Med. 2008 Mar-Apr;14(3-4):175-83. doi: 10.2119/2007-00095.Alleva.
  3. Altunina NV, Lizogub VG, Bondarchuk OM. Alpha-Lipoic Acid as a Means of Influence on Systemic Inflammation in Type 2 Diabetes Mellitus Patients with Prior Myocardial Infarction. J Med Life. 2020 Jan-Mar;13(1):32-36. doi: 10.25122/jml-2020-0018.
  4. Barbosa NG, Gonzaga AKG, de Sena Fernandes LL, da Fonseca AG, Queiroz SIML, Lemos TMAM, da Silveira ÉJD, de Medeiros AMC. Evaluation of laser therapy and alpha-lipoic acid for the treatment of burning mouth syndrome: a randomized clinical trial. Lasers Med Sci. 2018 Aug;33(6):1255-1262. doi: 10.1007/s10103-018-2472-2.
  5. Bertolotto F, Massone A. Combination of alpha lipoic acid and superoxide dismutase leads to physiological and symptomatic improvements in diabetic neuropathy. Drugs R D. 2012 Mar 1;12(1):29-34. doi: 10.2165/11599200-000000000-00000.
  6. Bharucha AE, Choi KM, Saw JJ, Gibbons SJ, Farrugia GF, Carlson DA, Zinsmeister AR. Effects of aspirin & simvastatin and aspirin, simvastatin, & lipoic acid on heme oxygenase-1 in healthy human subjects. Neurogastroenterol Motil. 2014 Oct;26(10):1437-42. doi: 10.1111/nmo.12404.
  7. Borcea V, Nourooz-Zadeh J, Wolff SP, Klevesath M, Hofmann M, Urich H, Wahl P, Ziegler R, Tritschler H, Halliwell B, Nawroth PP. alpha-Lipoic acid decreases oxidative stress even in diabetic patients with poor glycemic control and albuminuria. Free Radic Biol Med. 1999 Jun;26(11-12):1495-500. doi: 10.1016/s0891-5849(99)00011-8. PMID: 10401614.
  8. Brennan BP, Jensen JE, Hudson JI, Coit CE, Beaulieu A, Pope HG Jr, Renshaw PF, Cohen BM. A placebo-controlled trial of acetyl-L-carnitine and α-lipoic acid in the treatment of bipolar depression. J Clin Psychopharmacol. 2013 Oct;33(5):627-35. doi: 10.1097/JCP.0b013e31829a83f5.
  9. Cameron M, Taylor C, Lapidus J, Ramsey K, Koop D, Spain R. Gastrointestinal Tolerability and Absorption of R- Versus R,S-Lipoic Acid in Progressive Multiple Sclerosis: A Randomized Crossover Trial. J Clin Pharmacol. 2020 Aug;60(8):1099-1106. doi: 10.1002/jcph.1605.
  10. Camporese G, Bernardi E, Bortoluzzi C, Kontothanassis D, Tonello C, Nardin M, Noventa F. Complex α-lipoic acid, ginkgoselect phytosome and leucoselect phytosome in patients with chronic venous insufficiency of the lower limbs: therapeutic effectiveness and impact on quality of life. J Blood Lymph. 2008;8:1. doi: 10.4172/2165-7831.1000210.
  11. Canepa P, Dal Lago A, De Leo C, Gallo M, Rizzo C, Licata E, Anserini P, Rago R, Scaruffi P. Combined treatment with myo-inositol, alpha-lipoic acid, folic acid and vitamins significantly improves sperm parameters of sub-fertile men: a multi-centric study. Eur Rev Med Pharmacol Sci. 2018 Oct;22(20):7078-7085. doi: 10.26355/eurrev_201810_16180.
  12. Cappelli V, Di Sabatino A, Musacchio MC, De Leo V. Valutazione di una nuova associazione tra insulino-sensibilizzanti e acido α-lipoico in donne obese affette da PCOS. Minerva Ginecol. 2013;65:425-433. Italian.
  13. Cavestro C, Bedogni G, Molinari F, Mandrino S, Rota E, Frigeri MC. Alpha-Lipoic Acid Shows Promise to Improve Migraine in Patients with Insulin Resistance: A 6-Month Exploratory Study. J Med Food. 2018 Mar;21(3):269-273. doi: 10.1089/jmf.2017.0068.
  14. Chang JW, Lee EK, Kim TH, Min WK, Chun S, Lee KU, Kim SB, Park JS. Effects of alpha-lipoic acid on the plasma levels of asymmetric dimethylarginine in diabetic end-stage renal disease patients on hemodialysis: a pilot study. Am J Nephrol. 2007;27(1):70-4. doi: 10.1159/000099035.
  15. Cicek M, Yıldırır A, Okyay K, Yazici AC, Aydinalp A, Kanyilmaz S, Muderrisoglu H. Use of alpha-lipoic acid in prevention of contrast-induced nephropathy in diabetic patients. Ren Fail. 2013;35(5):748-53. doi: 10.3109/0886022X.2013.790298.
  16. De Cicco S, Immediata V, Romualdi D, Policola C, Tropea A, Di Florio C, Tagliaferri V, Scarinci E, Della Casa S, Lanzone A, Apa R. Myoinositol combined with alpha-lipoic acid may improve the clinical and endocrine features of polycystic ovary syndrome through an insulin-independent action. Gynecol Endocrinol. 2017 Sep;33(9):698-701. doi: 10.1080/09513590.2017.1313972.
  17. De Leo V, Cagnacci A, Cappelli V, Biasioli A, Leonardi D, Seracchioli R. Role of a natural integrator based on lipoic acid, palmitoiletanolamide and myrrh in the treatment of chronic pelvic pain and endometriosis. Minerva Ginecol. 2019 Jun;71(3):191-195. doi: 10.23736/S0026-4784.19.04384-3.
  18. Di Nicuolo F, D'Ippolito S, Castellani R, Rossi ED, Masciullo V, Specchia M, Mariani M, Pontecorvi A, Scambia G, Di Simone N. Effect of alpha-lipoic acid and myoinositol on endometrial inflammasome from recurrent pregnancy loss women. Am J Reprod Immunol. 2019 Sep;82(3):e13153. doi: 10.1111/aji.13153.
  19. Dworacka M, Iskakova S, Krzyżagórska E, Wesołowska A, Kurmambayev Y, Dworacki G. Alpha-lipoic acid modifies circulating angiogenic factors in patients with type 2 diabetes mellitus. Diabetes Res Clin Pract. 2015 Feb;107(2):273-9. doi: 10.1016/j.diabres.2014.11.005.
  20. Emsley R, Chiliza B, Asmal L, du Plessis S, Phahladira L, van Niekerk E, van Rensburg SJ, Harvey BH. A randomized, controlled trial of omega-3 fatty acids plus an antioxidant for relapse prevention after antipsychotic discontinuation in first-episode schizophrenia. Schizophr Res. 2014 Sep;158(1-3):230-5. doi: 10.1016/j.schres.2014.06.004.
  21. Femiano F, Gombos F, Scully C. Burning mouth syndrome: the efficacy of lipoic acid on subgroups. J Eur Acad Dermatol Venereol. 2004 Nov;18(6):676-8. doi: 10.1111/j.1468-3083.2004.01049.x.
  22. Fruzzetti F, Capozzi A, Canu A, Lello S. Treatment with d-chiro-inositol and alpha lipoic acid in the management of polycystic ovary syndrome. Gynecol Endocrinol. 2019 Jun;35(6):506-510. doi: 10.1080/09513590.2018.1540573.
  23. Gębka A, Serkies-Minuth E, Raczyńska D. Effect of the administration of alpha-lipoic acid on contrast sensitivity in patients with type 1 and type 2 diabetes. Mediators Inflamm. 2014;2014:131538. doi: 10.1155/2014/131538. Epub 2014 Feb 10.
  24. Genazzani AD, Prati A, Marchini F, Petrillo T, Napolitano A, Simoncini T. Differential insulin response to oral glucose tolerance test (OGTT) in overweight/obese polycystic ovary syndrome patients undergoing to myo-inositol (MYO), alpha lipoic acid (ALA), or combination of both. Gynecol Endocrinol. 2019 Dec;35(12):1088-1093. doi: 10.1080/09513590.2019.1640200.
  25. Genazzani AD, Shefer K, Della Casa D, Prati A, Napolitano A, Manzo A, Despini G, Simoncini T. Modulatory effects of alpha-lipoic acid (ALA) administration on insulin sensitivity in obese PCOS patients. J Endocrinol Invest. 2018 May;41(5):583-590. doi: 10.1007/s40618-017-0782-z.
  26. Hegazy SK, Tolba OA, Mostafa TM, Eid MA, El-Afify DR. Alpha-lipoic acid improves subclinical left ventricular dysfunction in asymptomatic patients with type 1 diabetes. Rev Diabet Stud. 2013 Spring;10(1):58-67. doi: 10.1900/RDS.2013.10.58. Epub 2013 May 10.
  27. Hong Y, Peng J, Cai X, Zhang X, Liao Y, Lan L. Clinical Efficacy of Alprostadil Combined with α-lipoic Acid in the Treatment of Elderly Patients with Diabetic Nephropathy. Open Med (Wars). 2017 Oct 5;12:323-327. doi: 10.1515/med-2017-0046.
  28. Hosseinpour-Arjmand S, Amirkhizi F, Ebrahimi-Mameghani M. The effect of alpha-lipoic acid on inflammatory markers and body composition in obese patients with non-alcoholic fatty liver disease: A randomized, double-blind, placebo-controlled trial. J Clin Pharm Ther. 2019 Apr;44(2):258-267. doi: 10.1111/jcpt.12784.
  29. Hummel T, Heilmann S, Hüttenbriuk KB. Lipoic acid in the treatment of smell dysfunction following viral infection of the upper respiratory tract. Laryngoscope. 2002 Nov;112(11):2076-80. doi: 10.1097/00005537-200211000-00031.
  30. Iezzi ML, Varriale G, Torge N, Lasorella S, Zagaroli L, Verrotti A. Polycystic ovary syndrome in adolescence: new therapeutic approach with inositol and alpha-lipoic acid. IJCR. July 2017;9(7):54006-54011.
  31. Jo SH, Kim SA, Kim HS, Han SJ, Park WJ, Choi YJ. Alpha-lipoic acid for the prevention of contrast-induced nephropathy in patients undergoing coronary angiography: the ALIVE study - a prospective randomized trial. Cardiology. 2013;126(3):159-66. doi: 10.1159/000353812.
  32. Kamenova P. Improvement of insulin sensitivity in patients with type 2 diabetes mellitus after oral administration of alpha-lipoic acid. Hormones (Athens). 2006 Oct-Dec;5(4):251-8. doi: 10.14310/horm.2002.11191.
  33. Konrad T, Vicini P, Kusterer K, Höflich A, Assadkhani A, Böhles HJ, Sewell A, Tritschler HJ, Cobelli C, Usadel KH. alpha-Lipoic acid treatment decreases serum lactate and pyruvate concentrations and improves glucose effectiveness in lean and obese patients with type 2 diabetes. Diabetes Care. 1999 Feb;22(2):280-7. doi: 10.2337/diacare.22.2.280.
  34. Lee SR, Jeong MH, Lim SY, Hong SN, Kim KH, Sohn IS, Jong YJ, Park HW, Kim HJ, Kim W, Ahn YK, Cho JG, Park JC, Kang JC. The Effect of Alpha Lipoic Acid (Thioctacid HR®) on Endothelial Function in Diabetic and Hypertensive Patients. Korean Circulation J. 2006;36:559-564.
  35. Letizia Mauro G, Cataldo P, Barbera G, Sanfilippo A. α-Lipoic acid and superoxide dismutase in the management of chronic neck pain: a prospective randomized study. Drugs R D. 2014 Mar;14(1):1-7. doi: 10.1007/s40268-013-0035-3.
  36. Marangon K, Devaraj S, Tirosh O, Packer L, Jialal I. Comparison of the effect of alpha-lipoic acid and alpha-tocopherol supplementation on measures of oxidative stress. Free Radic Biol Med. 1999 Nov;27(9-10):1114-21. doi: 10.1016/s0891-5849(99)00155-0.
  37. Memeo A, Loiero M. Thioctic acid and acetyl-L-carnitine in the treatment of sciatic pain caused by a herniated disc: a randomized, double-blind, comparative study. Clin Drug Investig. 2008;28(8):495-500. doi: 10.2165/00044011-200828080-00004.
  38. Minniti V, Pietris G, Barbullushi E, Meccoli G, Veltri A, Patore P. Usage of Ginkgo biloba and lipoic acid in patients with diabetic ulcers. Acta Vulnol. 2015;13:145-155.
  39. Mohammadshahi M, Zakizadeh E, Ahmadi-Angali K, Ravanbakhsh M, Helli B. The synergic effects of alpha-lipoic acid supplementation and electrical isotonic contraction on anthropometric measurements and the serum levels of VEGF, NO, sirtuin-1, and PGC1-α in obese people undergoing a weight loss diet. Arch Physiol Biochem. 2020 May 14:1-7. doi: 10.1080/13813455.2020.1762660.
  40. Morcos M, Borcea V, Isermann B, Gehrke S, Ehret T, Henkels M, Schiekofer S, Hofmann M, Amiral J, Tritschler H, Ziegler R, Wahl P, Nawroth PP. Effect of alpha-lipoic acid on the progression of endothelial cell damage and albuminuria in patients with diabetes mellitus: an exploratory study. Diabetes Res Clin Pract. 2001 Jun;52(3):175-83. doi: 10.1016/s0168-8227(01)00223-6.
  41. Morgante G, Cappelli V, Di Sabatino A, Massaro MG, De Leo V. Polycystic ovary syndrome (PCOS) and hyperandrogenism: the role of a new natural association. Minerva Ginecol. 2015 Oct;67(5):457-63.
  42. Nasole E, Nicoletti C, Yang ZJ, Girelli A, Rubini A, Giuffreda F, Di Tano A, Camporesi E, Bosco G. Effects of alpha lipoic acid and its R+ enantiomer supplemented to hyperbaric oxygen therapy on interleukin-6, TNF-α and EGF production in chronic leg wound healing. J Enzyme Inhib Med Chem. 2014 Apr;29(2):297-302. doi: 10.3109/14756366.2012.759951.
  43. Notarnicola A, Maccagnano G, Tafuri S, Fiore A, Pesce V, Moretti B. Comparison of shock wave therapy and nutraceutical composed of Echinacea angustifolia, alpha lipoic acid, conjugated linoleic acid and quercetin (perinerv) in patients with carpal tunnel syndrome. Int J Immunopathol Pharmacol. 2015 Jun;28(2):256-62. doi: 10.1177/0394632015584501.
  44. Okanović A, Prnjavorac B, Jusufović E, Sejdinović R. Alpha-lipoic acid reduces body weight and regulates triglycerides in obese patients with diabetes mellitus. Med Glas (Zenica). 2015 Aug;12(2):122-7. doi: 10.17392/798-15.
  45. Okuyucu S, Kocer A, Eryilmaz M, Kocaman G. Thioctacid is effective for neuropathy symptoms and hyperglycemia control without pronounced electromyographic changes. Neurosciences (Riyadh). 2012 Oct;17(4):384-6.
  46. Panda P, Panda SK, Mishra T. Effect of alpha lipoic acid in treatment of type 2 diabetes. Asian J Pharm Clin Res. 2017;10(8):204-7.
  47. Popa AR, Bungau S, Vesa CM, Bondar AC, Pantis C, Maghiar O, Dimulescu IA, Nistor Cseppento CA, Rus M. Evaluating the Efficacy of the Treatment with Benfotiamine and Alpha-lipoic Acid in Distal Symmetric Painful Diabetic Polyneuropathy. Rev Chim (Buchar). 2019;70(9): 3108-14.
  48. Rahman ST, Merchant N, Haque T, Wahi J, Bhaheetharan S, Ferdinand KC, Khan BV. The impact of lipoic acid on endothelial function and proteinuria in quinapril-treated diabetic patients with stage I hypertension: results from the QUALITY study. J Cardiovasc Pharmacol Ther. 2012 Jun;17(2):139-45. doi: 10.1177/1074248411413282.
  49. Rago R, Marcucci I, Leto G, Caponecchia L, Salacone P, Bonanni P, Fiori C, Sorrenti G, Sebastianelli A. Effect of myo-inositol and alpha-lipoic acid on oocyte quality in polycystic ovary syndrome non-obese women undergoing in vitro fertilization: a pilot study. J Biol Regul Homeost Agents. 2015 Oct-Dec;29(4):913-23.
  50. Ramos LF, Kane J, McMonagle E, Le P, Wu P, Shintani A, Ikizler TA, Himmelfarb J. Effects of combination tocopherols and alpha lipoic acid therapy on oxidative stress and inflammatory biomarkers in chronic kidney disease. J Ren Nutr. 2011 May;21(3):211-8. doi: 10.1053/j.jrn.2010.08.003.
  51. Ranieri M, Sciuscio M, Cortese AM, Santamato A, Di Teo L, Ianieri G, Bellomo RG, Stasi M, Megna M. The use of alpha-lipoic acid (ALA), gamma linolenic acid (GLA) and rehabilitation in the treatment of back pain: effect on health-related quality of life. Int J Immunopathol Pharmacol. 2009 Jul-Sep;22(3 Suppl):45-50. doi: 10.1177/03946320090220S309.
  52. Reboa G, Gipponi M, Fregatti P, Depaoli F. Integrated Treatment With Stapled Haemorrhoidopexy and Proctonorm® of Haemorrhoidal Disease. In Vivo. 2019 Sep-Oct;33(5):1671-1675. doi: 10.21873/invivo.11654.
  53. Roesler R, Quevedo J, Walz R, Bianchin M. A randomized, double-blind, placebo-controlled trial of deprenyl and thiotic acid in HIV-associated cognitive impairment. Neurology. 1999 Jun 10;52(9):1920-1.
  54. Šabanović M, Jašić M, Odobašić A, Aleksovska ES, Pavljašević S, Bajraktarević A, Čepo DV. Alpha Lipoic Acid Reduces Symptoms and Inflammation Biomarkers in Patients with Chronic Hemorrhoidal Illness. Int J Vitam Nutr Res. 2018 Dec;88(5-6):281-290. doi: 10.1024/0300-9831/a000251.
  55. Shinto L, Quinn J, Montine T, Dodge HH, Woodward W, Baldauf-Wagner S, Waichunas D, Bumgarner L, Bourdette D, Silbert L, Kaye J. A randomized placebo-controlled pilot trial of omega-3 fatty acids and alpha lipoic acid in Alzheimer's disease. J Alzheimers Dis. 2014;38(1):111-20. doi: 10.3233/JAD-130722.
  56. Solakovic S, Solakovic E, Pavlovic R, Ahmovic A, Vrcic M, Covic Nedim. The Comparison of Stationary Bike Training and Standard Treadmill Therapy in Overweight and Obese Patients Age 40-55 with Peripheral Artery Disease (PAD) in Fontaine’s Stage IIa on High Dose Supplementation Support of Alpha-Lipoic Acid (ALA). J Sport Sci Med. 2019;7(2):51-60. doi: 10.12691/ajssm-7-2-5
  57. Sosa-Estani S, Armenti A, Araujo G, Viotti R, Lococo B, Ruiz Vera B, Vigliano C, de Rissio AM, Segura EL. Tratamiento de la enfermedad de Chagas con benznidazol y ácido tióctico [Treatment of Chagas disease with benznidazole and thioctic acid]. Medicina (B Aires). 2004;64(1):1-6. Spanish.
  58. Tankova T, Koev D, Dakovska L. Alpha-lipoic acid in the treatment of autonomic diabetic neuropathy (controlled, randomized, open-label study). Rom J Intern Med. 2004;42(2):457-64.
  59. Usta Atmaca H, Akbas F. THE EFFECT OF SHORT TERM ALPHA LIPOIC ACID ADMINISTRATION ON ADIPONECTIN AND BODY WEIGHT IN TYPE 2 DIABETES MELLITUS PATIENTS. Acta Endocrinol (Buchar). 2017 Oct-Dec;13(4):461-466. doi: 10.4183/aeb.2017.461.
  60. Vidović B, Milovanović S, Stefanović A, Kotur-Stevuljević J, Takić M, Debeljak-Martačić J, Pantović M, Đorđević B. Effects of Alpha-Lipoic Acid Supplementation on Plasma Adiponectin Levels and Some Metabolic Risk Factors in Patients with Schizophrenia. J Med Food. 2017 Jan;20(1):79-85. doi: 10.1089/jmf.2016.0070.
  61. Vidović B, Milovanović S, Dorđević B, Kotur-Stevuljević J, Stefanović A, Ivanišević J, Miljković M, Spasić S, Stojanović D, Pantović M. Effect of alpha-lipoic acid supplementation on oxidative stress markers and antioxidative defense in patients with schizophrenia. Psychiatr Danub. 2014 Sep;26(3):205-13.
  62. Won JC, Kwon HS, Moon SS, Chun SW, Kim CH, Park IB, Kim IJ, Lee J, Cha BY, Park TS. γ-Linolenic Acid versus α-Lipoic Acid for Treating Painful Diabetic Neuropathy in Adults: A 12-Week, Double-Placebo, Randomized, Noninferiority Trial. Diabetes Metab J. 2019 Nov 4. doi: 10.4093/dmj.2019.0099.
  63. Xiao C, Giacca A, Lewis GF. Short-term oral α-lipoic acid does not prevent lipid-induced dysregulation of glucose homeostasis in obese and overweight nondiabetic men. Am J Physiol Endocrinol Metab. 2011 Oct;301(4):E736-41. doi: 10.1152/ajpendo.00183.2011.
  64. Yücel M, Taşdemir S, Çetiz A, Akgün H, Taşlipinar A, Erdoğan C, Ulaş UH, Odabaşi Z, Kütükçü Y. Effect of Alpha-Lipoic Acid on Small Fibre Neuropathy Findings in Patients With Type 2 Diabetes Mellitus. JNS. 2015;32(2):311-319.
- **Lack of randomization (n= 5)**
  1. Femiano F, Gombos F, Scully C. Burning Mouth Syndrome: open trial of psychotherapy alone, medication with alpha-lipoic acid (thioctic acid), and combination therapy. Med Oral. 2004 Jan-Feb;9(1):8-13. English, Spanish.
  2. Femiano F, Scully C, Gombos F. Idiopathic dysgeusia; an open trial of alpha lipoic acid (ALA) therapy. Int J Oral Maxillofac Surg. 2002 Dec;31(6):625-8. doi: 10.1054/ijom.2002.0276.
  3. Femiano F. Burning mouth syndrome (BMS): an open trial of comparative efficacy of alpha-lipoic acid (thioctic acid) with other therapies. Minerva Stomatol. 2002 Sep;51(9):405-9.
  4. Femiano F, Gombos F, Scully C, Busciolano M, De Luca P. Burning mouth syndrome (BMS): controlled open trial of the efficacy of alpha-lipoic acid (thioctic acid) on symptomatology. Oral Dis. 2000 Sep;6(5):274-7. doi: 10.1111/j.1601-0825.2000.tb00138.x.
  5. Gamidov SI, Ovchinnikov RI, Popova AY. [Double-blind, randomized placebo-controlled study of efficiency and safety of complex acetyl-L-carnitine, L-carnitine fumarate and alpha-lipoic acid (Spermactin Forte) for treatment of male infertility]. Urologiia. 2019 Sep;(4):62-68. Russian.
- **Acute effect of α-lipoic acid supplementation (n= 7)**
  1. Casciato P, Ambrosi N, Caro F, Vazquez M, Müllen E, Gadano A, de Santibañes E, de Santibañes M, Zandomeni M, Chahdi M, Lazarte JC, Biagiola DA, Iaquinandi JC, Santofimia-Castaño P, Iovanna J, Incardona C, Chuluyan E. α-lipoic acid reduces postreperfusion syndrome in human liver transplantation - a pilot study. Transpl Int. 2018 Dec;31(12):1357-1368. doi: 10.1111/tri.13314.
  2. Sharman JE, Gunaruwan P, Knez WL, Schmitt M, Marsh SA, Wilson GR, Cockcroft JR, Coombes JS. Alpha-lipoic acid does not acutely affect resistance and conduit artery function or oxidative stress in healthy men. Br J Clin Pharmacol. 2004 Sep;58(3):243-8. doi: 10.1111/j.1365-2125.2004.02146.x.
  3. Smiljanec K, Mbakwe AU, Ramos-Gonzalez M, Pohlig RT, Lennon SL. Antioxidant cocktail following a high-sodium meal does not affect vascular function in young, healthy adult humans: a randomized controlled crossover trial. Nutr Res. 2020 May 24;79:13-22. doi: 10.1016/j.nutres.2020.05.011.
  4. Tcheng JE, Gibson M, Krucoff MW, Patel MR, Ajit M, Hiremath J, Ponde C, Ramsaran E, Clark G, Lader AS, Beeuwkes R 3rd. SUPPORT-1 (Subjects Undergoing PCI and Perioperative Reperfusion Treatment): A Prospective, Randomized Trial of CMX-2043 in Patients Undergoing Elective Percutaneous Coronary Intervention. J Cardiovasc Pharmacol. 2020 Aug;76(2):189-196. doi: 10.1097/FJC.0000000000000830.
  5. Vucinovic Z, Duplancic D, Seselja-Perisin A, Kukoc-Modun L, Gunjaca G, Radman M, Vukovic J, Tsikas D, Poljak K, Modun D. Acute application of antioxidants protects against hyperoxia-induced reduction of plasma nitrite concentration. Clin Physiol Funct Imaging. 2015 Jan;35(1):76-80. doi: 10.1111/cpf.12169.
  6. Wray DW, Nishiyama SK, Harris RA, Zhao J, McDaniel J, Fjeldstad AS, Witman MA, Ives SJ, Barrett-O'Keefe Z, Richardson RS. Acute reversal of endothelial dysfunction in the elderly after antioxidant consumption. Hypertension. 2012 Apr;59(4):818-24. doi: 10.1161/HYPERTENSIONAHA.111.189456.
  7. Xiang GD, Sun HL, Zhao LS, Hou J, Yue L, Xu L. The antioxidant alpha-lipoic acid improves endothelial dysfunction induced by acute hyperglycaemia during OGTT in impaired glucose tolerance. Clin Endocrinol (Oxf). 2008 May;68(5):716-23. doi: 10.1111/j.1365-2265.2007.03099.x.
- **Studies testing α-lipoic acid supplementation combined in nutraceutical compounds (n= 27)**
  1. Amirkhizi F, Hamedi-Shahraki S, Hosseinpour-Arjmand S, Vaghef-Mehrabany E, Ebrahimi-Mameghani M. Effects of Alpha-Lipoic Acid Supplementation on Oxidative Stress Status in Patients with Non-Alcoholic Fatty Liver Disease: A Randomized, Double-Blind, Placebo-Controlled Clinical Trial, Iran Red Crescent Med J. 2018 ; 20(9):e67615. doi: 10.5812/ircmj.67615.
  2. Anwar AA, Tabri F, Adriani a, Djawad K, Bukhari A, Patellongi I. The effect of glutathione supplementation (l-glutathione, vitamin c, alpha lipoic acid, and zinc) on total antioxidant status (TAS) level. Int J Med Rev Case Rep. 2019. [In Press]
  3. Baillie JK, Thompson AA, Irving JB, Bates MG, Sutherland AI, Macnee W, Maxwell SR, Webb DJ. Oral antioxidant supplementation does not prevent acute mountain sickness: double blind, randomized placebo-controlled trial. QJM. 2009 May;102(5):341-8. doi: 10.1093/qjmed/hcp026.
  4. Bailey DM, Davies B. Acute mountain sickness; prophylactic benefits of antioxidant vitamin supplementation at high altitude. High Alt Med Biol. 2001 Spring;2(1):21-9. doi: 10.1089/152702901750067882.
  5. Block G, Jensen C, Dietrich M, Norkus EP, Hudes M, Packer L. Plasma C-reactive protein concentrations in active and passive smokers: influence of antioxidant supplementation. J Am Coll Nutr. 2004 Apr;23(2):141-7. doi: 10.1080/07315724.2004.10719354.
  6. Capasso I, Esposito E, Maurea N, Montella M, Crispo A, De Laurentiis M, D'Aiuto M, Frasci G, Botti G, Grimaldi M, Cavalcanti E, Esposito G, Fucito A, Brillante G, D'Aiuto G, Ciliberto G. Combination of inositol and alpha lipoic acid in metabolic syndrome-affected women: a randomized placebo-controlled trial. Trials. 2013 Aug 28;14:273. doi: 10.1186/1745-6215-14-273.
  7. Cicero AFG, Fogacci F, Morbini M, Colletti A, Bove M, Veronesi M, Giovannini M, Borghi C. Nutraceutical Effects on Glucose and Lipid Metabolism in Patients with Impaired Fasting Glucose: A Pilot, Double-Blind, Placebo-Controlled, Randomized Clinical Trial on a Combined Product. High Blood Press Cardiovasc Prev. 2017 Sep;24(3):283-288. doi: 10.1007/s40292-017-0206-3.
  8. Costanzo L, Grasso A, Tamburino C, Di Pino L. Usefulness of a-lipoic acid, leucoselect and ginkgoselect phytosoma in chronic venous insufficiency. Angiol. 2016;4:2. doi: 10.4172/2329-9495.1000174.
  9. Dell'Anna ML, Mastrofrancesco A, Sala R, Venturini M, Ottaviani M, Vidolin AP, Leone G, Calzavara PG, Westerhof W, Picardo M. Antioxidants and narrow band-UVB in the treatment of vitiligo: a double-blind placebo controlled trial. Clin Exp Dermatol. 2007 Nov;32(6):631-6. doi: 10.1111/j.1365-2230.2007.02514.x. PMID: 17953631.
  10. de Oliveira AM, Rondó PH, Luzia LA, D'Abronzo FH, Illison VK. The effects of lipoic acid and α-tocopherol supplementation on the lipid profile and insulin sensitivity of patients with type 2 diabetes mellitus: a randomized, double-blind, placebo-controlled trial. Diabetes Res Clin Pract. 2011 May;92(2):253-60. doi: 10.1016/j.diabres.2011.02.010.
  11. Derosa G, D'Angelo A, Romano D, Maffioli P. A Clinical Trial about a Food Supplement Containing α-Lipoic Acid on Oxidative Stress Markers in Type 2 Diabetic Patients. Int J Mol Sci. 2016 Oct 28;17(11):1802. doi: 10.3390/ijms17111802.
  12. Gabbay E, Zigmond E, Pappo O, Hemed N, Rowe M, Zabrecky G, Cohen R, Ilan Y. Antioxidant therapy for chronic hepatitis C after failure of interferon: results of phase II randomized, double-blind placebo controlled clinical trial. World J Gastroenterol. 2007 Oct 28;13(40):5317-23. doi: 10.3748/wjg.v13.i40.5317.
  13. Galasko DR, Peskind E, Clark CM, Quinn JF, Ringman JM, Jicha GA, Cotman C, Cottrell B, Montine TJ, Thomas RG, Aisen P; Alzheimer’s Disease Cooperative Study. Antioxidants for Alzheimer disease: a randomized clinical trial with cerebrospinal fluid biomarker measures. Arch Neurol. 2012 Jul;69(7):836-41. doi: 10.1001/archneurol.2012.85.
  14. Kaiser JD, Campa AM, Ondercin JP, Leoung GS, Pless RF, Baum MK. Micronutrient supplementation increases CD4 count in HIV-infected individuals on highly active antiretroviral therapy: a prospective, double-blinded, placebo-controlled trial. J Acquir Immune Defic Syndr. 2006 Aug 15;42(5):523-8. doi: 10.1097/01.qai.0000230529.25083.42.
  15. Karkabounas S, Papadopoulos N, Anastasiadou C, Gubili C, Peschos D, Daskalou T, Fikioris N, Simos YV, Kontargiris E, Gianakopoulos X, Ragos V, Chatzidimitriou M. Effects of α-Lipoic Acid, Carnosine, and Thiamine Supplementation in Obese Patients with Type 2 Diabetes Mellitus: A Randomized, Double-Blind Study. J Med Food. 2018 Dec;21(12):1197-1203. doi: 10.1089/jmf.2018.0007.
  16. Lott IT, Doran E, Nguyen VQ, Tournay A, Head E, Gillen DL. Down syndrome and dementia: a randomized, controlled trial of antioxidant supplementation. Am J Med Genet A. 2011 Aug;155A(8):1939-48. doi: 10.1002/ajmg.a.34114.
  17. McMackin CJ, Widlansky ME, Hamburg NM, Huang AL, Weller S, Holbrook M, Gokce N, Hagen TM, Keaney JF Jr, Vita JA. Effect of combined treatment with alpha-Lipoic acid and acetyl-L-carnitine on vascular function and blood pressure in patients with coronary artery disease. J Clin Hypertens (Greenwich). 2007 Apr;9(4):249-55. doi: 10.1111/j.1524-6175.2007.06052.x.
  18. Nebbioso M, Federici M, Rusciano D, Evangelista M, Pescosolido N. Oxidative stress in preretinopathic diabetes subjects and antioxidants. Diabetes Technol Ther. 2012 Mar;14(3):257-63. doi: 10.1089/dia.2011.0172.
  19. Noori N, Tabibi H, Hosseinpanah F, Hedayati M, Nafar M. Effects of combined lipoic acid and pyridoxine on albuminuria, advanced glycation end-products, and blood pressure in diabetic nephropathy. Int J Vitam Nutr Res. 2013;83(2):77-85. doi: 10.1024/0300-9831/a000147.
  20. Parravano M, Tedeschi M, Manca D, Costanzo E, Di Renzo A, Giorno P, Barbano L, Ziccardi L, Varano M, Parisi V. Effects of Macuprev® Supplementation in Age-Related Macular Degeneration: A Double-Blind Randomized Morpho-Functional Study Along 6 Months of Follow-Up. Adv Ther. 2019 Sep;36(9):2493-2505. doi: 10.1007/s12325-019-01016-2. Epub 2019 Jun 25. Erratum in: Adv Ther. 2019 Nov;36(11):3288.
  21. Polanski JF, Soares AD, de Mendonça Cruz OL. Antioxidant therapy in the elderly with tinnitus. Braz J Otorhinolaryngol. 2016 May-Jun;82(3):269-74. doi: 10.1016/j.bjorl.2015.04.016.
  22. Polanski JF, Cruz OL. Evaluation of antioxidant treatment in presbyacusis: prospective, placebo-controlled, double-blind, randomised trial. J Laryngol Otol 2013 02;127(2):134-41.
  23. Pop-Busui R, Stevens MJ, Raffel DM, White EA, Mehta M, Plunkett CD, Brown MB, Feldman EL. Effects of triple antioxidant therapy on measures of cardiovascular autonomic neuropathy and on myocardial blood flow in type 1 diabetes: a randomised controlled trial. Diabetologia. 2013 Aug;56(8):1835-44. doi: 10.1007/s00125-013-2942-9.
  24. Richer S, Stiles W, Statkute L, Pulido J, Frankowski J, Rudy D, Pei K, Tsipursky M, Nyland J. Double-masked, placebo-controlled, randomized trial of lutein and antioxidant supplementation in the intervention of atrophic age-related macular degeneration: the Veterans LAST study (Lutein Antioxidant Supplementation Trial). Optometry. 2004 Apr;75(4):216-30. doi: 10.1016/s1529-1839(04)70049-4.
  25. Thom E. A randomized, double-blind, placebo-controlled study on the clinical efficacy of oral treatment with DermaVite on ageing symptoms of the skin. J Int Med Res. 2005 May-Jun;33(3):267-72. doi: 10.1177/147323000503300301.
  26. Udupa AS, Nahar PS, Shah SH, Kshirsagar MJ, Ghongane BB. Study of comparative effects of antioxidants on insulin sensitivity in type 2 diabetes mellitus. J Clin Diagn Res. 2012 Nov;6(9):1469-73. doi: 10.7860/JCDR/2012/4464.2535.
  27. Yu AL, Moraru O, Erb C, Welge-Lussen U. Effects of Micronutrients on Serum Antioxidant Status of Glaucoma Patients: A Randomized, Placebo-controlled, Double-masked Pilot Study. OR. 2014;2(1):1-9.
- **Intravenous treatment with α-lipoic acid (n= 11)**
  1. Ali RM, Khairy MA, Mansour DY. Effect of alpha-lipoic acid on acute lung injury and acute kidney injury in major postpartum hemorrhage. Ain-Shams J Anaesthesiol 2016;9:219-24.
  2. Ametov AS, Novosadova MV, Barinov AN, Samigullin R, Trischler HJ. [Long-term effect of 3-week intravenous alpha-lipoic acid administration in symptomatic diabetic polyneutropathy with clinical manifestations]. Ter Arkh. 2010;82(12):61-4. Russian.
  3. Ametov AS, Barinov A, Dyck PJ, Hermann R, Kozlova N, Litchy WJ, Low PA, Nehrdich D, Novosadova M, O'Brien PC, Reljanovic M, Samigullin R, Schuette K, Strokov I, Tritschler HJ, Wessel K, Yakhno N, Ziegler D; SYDNEY Trial Study Group. The sensory symptoms of diabetic polyneuropathy are improved with alpha-lipoic acid: the SYDNEY trial. Diabetes Care. 2003 Mar;26(3):770-6. doi: 10.2337/diacare.26.3.770. Erratum in: Diabetes Care. 2003 Jul;26(7):2227.
  4. Heinisch BB, Francesconi M, Mittermayer F, Schaller G, Gouya G, Wolzt M, Pleiner J. Alpha-lipoic acid improves vascular endothelial function in patients with type 2 diabetes: a placebo-controlled randomized trial. Eur J Clin Invest. 2010 Feb;40(2):148-54. doi: 10.1111/j.1365-2362.2009.02236.x.
  5. Mittermayer F, Pleiner J, Francesconi M, Wolzt M. Treatment with alpha-lipoic acid reduces asymmetric dimethylarginine in patients with type 2 diabetes mellitus. Transl Res. 2010 Jan;155(1):6-9. doi: 10.1016/j.trsl.2009.08.004.
  6. Reljanovic M, Reichel G, Rett K, Lobisch M, Schuette K, Möller W, Tritschler HJ, Mehnert H. Treatment of diabetic polyneuropathy with the antioxidant thioctic acid (alpha-lipoic acid): a two year multicenter randomized double-blind placebo-controlled trial (ALADIN II). Alpha Lipoic Acid in Diabetic Neuropathy. Free Radic Res. 1999 Sep;31(3):171-9. doi: 10.1080/10715769900300721.
  7. Strokov IA, Kozlova NA, Mozolevskiĭ IuV, Miasoedov SP, Iakhno NN. Effektivnost' vnutrivennogo vvedeniia trometamolovoĭ soli tioktovoĭ (al'fa-lipoevoĭ) kisloty pri diabeticheskoĭ nevropatii [The efficacy of the intravenous administration of the trometamol salt of thioctic (alpha-lipoic) acid in diabetic neuropathy]. Zh Nevrol Psikhiatr Im S S Korsakova. 1999;99(6):18-22. Russian.
  8. Veresiu IA. Intravenous alpha-lipoic acid improves fructosamine level in type 2 diabetes patients. Acta Endocrinol (Buchar). 2009;5(4):501-506. doi: 10.4183/acb.2009.501
  9. Zhao L, Hu FX. α-Lipoic acid treatment of aged type 2 diabetes mellitus complicated with acute cerebral infarction. Eur Rev Med Pharmacol Sci. 2014;18(23):3715-9.
  10. Ziegler D, Hanefeld M, Ruhnau KJ, Hasche H, Lobisch M, Schütte K, Kerum G, Malessa R. Treatment of symptomatic diabetic polyneuropathy with the antioxidant alpha-lipoic acid: a 7-month multicenter randomized controlled trial (ALADIN III Study). ALADIN III Study Group. Alpha-Lipoic Acid in Diabetic Neuropathy. Diabetes Care. 1999 Aug;22(8):1296-301. doi: 10.2337/diacare.22.8.1296.
  11. Ziegler D, Hanefeld M, Ruhnau KJ, Meissner HP, Lobisch M, Schütte K, Gries FA. Treatment of symptomatic diabetic peripheral neuropathy with the anti-oxidant alpha-lipoic acid. A 3-week multicentre randomized controlled trial (ALADIN Study). Diabetologia. 1995 Dec;38(12):1425-33. doi: 10.1007/BF00400603.
- **Topical treatment with α-lipoic acid (n= 4)**
  1. Beitner H. Randomized, placebo-controlled, double blind study on the clinical efficacy of a cream containing 5% alpha-lipoic acid related to photoageing of facial skin. Br J Dermatol. 2003 Oct;149(4):841-9. doi: 10.1046/j.1365-2133.2003.05597.x.
  2. Grandi G, Pignatti L, Ferrari F, Dante G, Neri I, Facchinetti F. Vaginal alpha-lipoic acid shows an anti-inflammatory effect on the cervix, preventing its shortening after primary tocolysis. A pilot, randomized, placebo-controlled study. J Matern Fetal Neonatal Med. 2017 Sep;30(18):2243-2249. doi: 10.1080/14767058.2016.1245282.
  3. Sherif S, Bendas ER, Badawy S. The clinical efficacy of cosmeceutical application of liquid crystalline nanostructured dispersions of alpha lipoic acid as anti-wrinkle. Eur J Pharm Biopharm. 2014 Feb;86(2):251-9. doi: 10.1016/j.ejpb.2013.09.008.
  4. Volchegorskiĭ IA, Rassokhina LM, Koliadich MI, Alekseev MI. [Comparative study of alpha-lipoic acid and mexidol effects on affective status, cognitive functions and quality of life in diabetes mellitus patients]. Eksp Klin Farmakol. 2011;74(11):17-23. Russian.
- **Lack of sufficient information about the nature of the adverse events (n= 9)**
  1. El-Nahas MR, Elkannishy G, Abdelhafez H, Elkhamisy ET, El-Sehrawy AA. Oral Alpha Lipoic Acid Treatment for Symptomatic Diabetic Peripheral Neuropathy: A Randomized Double-Blinded Placebo-Controlled Study. Endocr Metab Immune Disord Drug Targets. 2020 May 5. doi: 10.2174/1871530320666200506081407.
  2. Gu XM, Zhang SS, Wu JC, Tang ZY, Lu ZQ, Li H, Liu C, Chen L, Ning G. [Efficacy and safety of high-dose α-lipoic acid in the treatment of diabetic polyneuropathy]. Zhonghua Yi Xue Za Zhi. 2010 Sep 21;90(35):2473-6. Chinese.
  3. Haritoglou C, Gerss J, Hammes HP, Kampik A, Ulbig MW; RETIPON Study Group. Alpha-lipoic acid for the prevention of diabetic macular edema. Ophthalmologica. 2011;226(3):127-37. doi: 10.1159/000329470.
  4. Huang YD, Li N, Zhang WG, Hu XJ, Wang Q, Wang CC, Xu RW, Yan K, Hou XY, Naerkeze, Wang XL, Yan WL. [The effect of oral alpha-lipoic acid in overweight/obese individuals on brachial-ankle pulse wave velocity and supine blood pressure: a randomized, crossover, double-blind, placebo-controlled trial]. Zhonghua Liu Xing Bing Xue Za Zhi. 2011 Mar;32(3):290-6. Chinese.
  5. Kolahi S, Mirtaheri E, Pourghasem Gargari B, Khabbazi A, Hajalilou M, Asghari-Jafarabadi M, Mesgari Abbasi M. Oral administration of alpha-lipoic acid did not affect lipid peroxidation and antioxidant biomarkers in rheumatoid arthritis patients. Int J Vitam Nutr Res. 2019 Jul;89(1-2):13-21. doi: 10.1024/0300-9831/a000550.
  6. Li N, Yan W, Hu X, Huang Y, Wang F, Zhang W, Wang Q, Wang X, Sun K. Effects of oral α-lipoic acid administration on body weight in overweight or obese subjects: a crossover randomized, double-blind, placebo-controlled trial. Clin Endocrinol (Oxf). 2017 May;86(5):680-687. doi: 10.1111/cen.13303.
  7. Mahdavi R, Khabbazi T, Safa J. Alpha lipoic acid supplementation improved antioxidant enzyme activities in hemodialysis patients. Int J Vitam Nutr Res. 2019 Sep;89(3-4):161-167. doi: 10.1024/0300-9831/a000552.
  8. Yousefi M, Kavianpour M, Hesami S, Rashidi Nooshabadi M, Khadem Haghighian H. Effect of alpha-lipoic acid at the combination with mefenamic acid in girls with primary dysmenorrhea: randomized, double-blind, placebo-controlled clinical trial. Gynecol Endocrinol. 2019 Sep;35(9):782-786. doi: 10.1080/09513590.2019.1590544.
  9. Ziegler D, Schatz H, Conrad F, Gries FA, Ulrich H, Reichel G. Effects of treatment with the antioxidant alpha-lipoic acid on cardiac autonomic neuropathy in NIDDM patients. A 4-month randomized controlled multicenter trial (DEKAN Study). Deutsche Kardiale Autonome Neuropathie. Diabetes Care. 1997 Mar;20(3):369-73. doi: 10.2337/diacare.20.3.369.
- **Data overlapped with another publication (n= 4)**
  1. Mirtaheri E, Gargari BP, Kolahi S, Dehghan P, Asghari-Jafarabadi M, Hajalilou M, Shakiba Novin Z, Mesgari Abbasi M. Effects of Alpha-Lipoic Acid Supplementation on Inflammatory Biomarkers and Matrix Metalloproteinase-3 in Rheumatoid Arthritis Patients. J Am Coll Nutr. 2015;34(4):310-7. doi: 10.1080/07315724.2014.910740.
  2. Mohammadi V, Khorvash F, Feizi A, Askari G. The effect of alpha-lipoic acid supplementation on anthropometric indices and food intake in patients who experienced stroke: A randomized, double-blind, placebo-controlled clinical trial. J Res Med Sci. 2017 Aug 16;22:98. doi: 10.4103/jrms.JRMS_1_17.
  3. Tang J, Wingerchuk DM, Crum BA, Rubin DI, Demaerschalk BM. Alpha-lipoic acid may improve symptomatic diabetic polyneuropathy. Neurologist. 2007 May;13(3):164-7. doi: 10.1097/01.nrl.0000263703.78318.2b.
  4. Ziegler D, Low PA, Freeman R, Tritschler H, Vinik AI. Predictors of improvement and progression of diabetic polyneuropathy following treatment with α-lipoic acid for 4 years in the NATHAN 1 trial. J Diabetes Complications. 2016 Mar;30(2):350-6. doi: 10.1016/j.jdiacomp.2015.10.018.
