# Supplementary material for: Safety Evaluation of α-Lipoic Acid Supplementation: A Systematic Review and Meta-Analysis of Randomized Placebo-Controlled Clinical Studies
Source: Antioxidants (Basel). 2020 Oct 19;9(10):1011. doi: 10.3390/antiox9101011 (PMC7603186; doi:10.3390/antiox9101011)
Supplement: Supplementary file 1 [file antioxidants-09-01011-s001.zip › Supplementary Materials_14092020.doc]

**Supplementary Materials**

**Figure S1.** Plot showing leave-one-out sensitivity analysis for the risk of gastrointestinal AEs following ALA supplementation *versus* placebo.

**Figure S2.** Funnel plot detailing publication bias for the risk of gastrointestinal AEs following ALA supplementation *versus* placebo.

**Figure S3.** Plot showing leave-one-out sensitivity analysis for the risk of neurological AEs following ALA supplementation *versus* placebo.

**Figure S4.** Funnel plot detailing publication bias for the risk of neurological AEs following ALA supplementation *versus* placebo.

**Figure S5.** Plot showing leave-one-out sensitivity analysis for the risk of psychiatric disorders following ALA supplementation *versus* placebo.

**Figure S6.** Funnel plot detailing publication bias for the risk of psychiatric disorders following ALA supplementation *versus* placebo.

**Figure S7.** Plot showing leave-one-out sensitivity analysis for the risk of musculoskeletal AEs following ALA supplementation *versus* placebo.

**Figure S8.** Funnel plot detailing publication bias for the risk of musculoskeletal AEs following ALA supplementation *versus* placebo.

**Figure S9.** Plot showing leave-one-out sensitivity analysis for the risk of skin AEs following ALA supplementation *versus* placebo.

**Figure S10.** Funnel plot detailing publication bias for the risk of skin AEs following ALA supplementation *versus* placebo.

**Figure S11.** Plot showing leave-one-out sensitivity analysis for the risk of infections following ALA supplementation *versus* placebo.

**Figure S12.** Funnel plot detailing publication bias for the risk of infections following ALA supplementation *versus* placebo.

**Figure S13.** Plot showing leave-one-out sensitivity analysis for the risk of CV system AEs following ALA supplementation *versus* placebo.

**Figure S14.** Funnel plot detailing publication bias for the risk of CV system AEs following ALA supplementation *versus* placebo.

**Figure S15.** Plot showing leave-one-out sensitivity analysis for the risk of hospitalization following ALA supplementation *versus* placebo.

**Figure S16.** Plot showing leave-one-out sensitivity analysis for the risk of death following ALA supplementation *versus* placebo.

**Figure S17.** Funnel plot detailing publication bias for the risk of death following ALA supplementation *versus* placebo.
